# A Chiral Lanthanide Tag for Stable and Rigid Attachment to Single Cysteine Residues in Proteins for NMR, EPR and Time‐Resolved Luminescence Studies

**DOI:** 10.1002/chem.202101143

**Published:** 2021-07-29

**Authors:** Iresha D. Herath, Colum Breen, Sarah H. Hewitt, Thomas R. Berki, Ahmad F. Kassir, Charlotte Dodson, Martyna Judd, Shereen Jabar, Nicholas Cox, Gottfried Otting, Stephen J. Butler

**Affiliations:** ^1^ Research School of Chemistry The Australian National University Canberra ACT 2605 Australia; ^2^ Department of Chemistry Loughborough University Epinal Way Loughborough LE11 3TU UK; ^3^ Department of Pharmacy & Pharmacology University of Bath Claverton Down Bath BA2 7AY UK

**Keywords:** EPR spectroscopy, FRET, lanthanoid tags, luminescence, NMR spectroscopy

## Abstract

A lanthanide‐binding tag site‐specifically attached to a protein presents a tool to probe the protein by multiple spectroscopic techniques, including nuclear magnetic resonance, electron paramagnetic resonance and time‐resolved luminescence spectroscopy. Here a new stable chiral Ln^III^ tag, referred to as **C12**, is presented for spontaneous and quantitative reaction with a cysteine residue to generate a stable thioether bond. The synthetic protocol of the tag is relatively straightforward, and the tag is stable for storage and shipping. It displays greatly enhanced reactivity towards selenocysteine, opening a route towards selective tagging of selenocysteine in proteins containing cysteine residues. Loaded with Tb^III^ or Tm^III^ ions, the **C12** tag readily generates pseudocontact shifts (PCS) in protein NMR spectra. It produces a relatively rigid tether between lanthanide and protein, which is beneficial for interpretation of the PCSs by single magnetic susceptibility anisotropy tensors, and it is suitable for measuring distance distributions in double electron–electron resonance experiments. Upon reaction with cysteine or other thiol compounds, the Tb^III^ complex exhibits a 100‐fold enhancement in luminescence quantum yield, affording a highly sensitive turn‐on luminescence probe for time‐resolved FRET assays and enzyme reaction monitoring.

## Graphical abstract

**Lanthanoid keys to proteins**: A stable chiral lanthanide(III) tag reacts spontaneously and quantitatively with a cysteine residue to generate a stable thioether bond. The lanthanide‐binding tag presents a tool to probe proteins using multiple spectroscopic techniques, including NMR, electron paramagnetic resonance and time‐resolved FRET experiments.

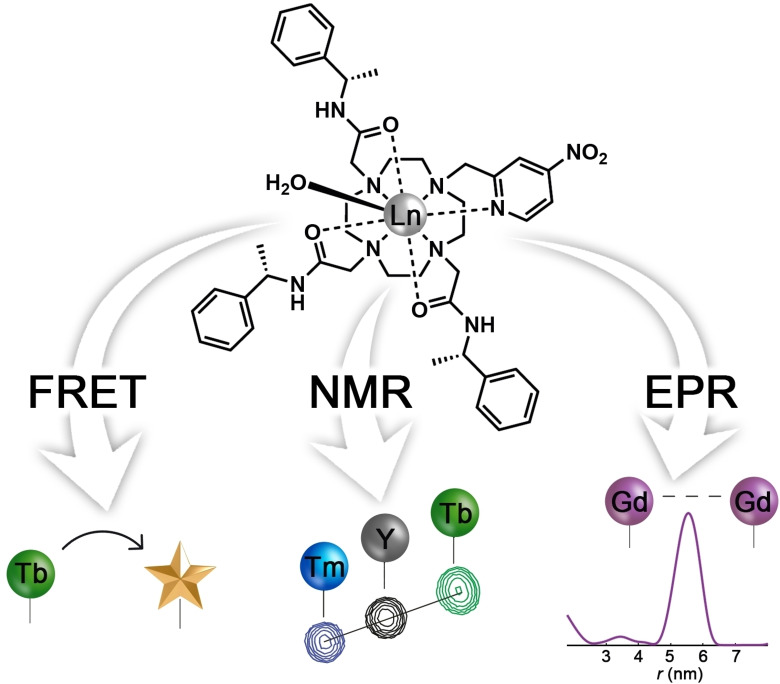

## Introduction

Site‐specific labelling of proteins with lanthanide complexes offers a powerful tool for a range of spectroscopic techniques, including nuclear magnetic resonance (NMR), electron paramagnetic resonance (EPR) and luminescence spectroscopy. Paramagnetic lanthanide ions produce large effects in protein NMR spectra that present long‐range structural restraints. Thus, paramagnetic relaxation enhancement (PRE) effects are observed for protons over 20 Å from a gadolinium(III) ion,^[1]^ pseudocontact shifts (PCS) are observable for nuclear spins over 40 Å from the paramagnetic centre^[2]^ and residual dipolar couplings (RDC) are observed throughout the entire molecule due to molecular alignment in the magnetic field conferred by a bound paramagnetic lanthanide ion (with the exception of Gd^III^).^[3]^

PCSs are of particular interest, as they are manifested in easy‐to‐measure changes in chemical shifts, which encode the position of the nuclear spin relative to the coordinate system defined by the magnetic susceptibility anisotropy (Δ*χ*) tensor associated with the paramagnetic centre. The PCS, *δ* ^PCS^, of a nuclear spin is given by

(1) $$\delta {}^{\mathrm{PCS}}=1/\left(12\;\pi r{}^{3}\right)\left[\Delta \chi {}_{\mathrm{ax}}\left(3\;\cos{}^{2}\theta -1\right)+3/2\;\Delta \chi {}_{\mathrm{rh}}\;\sin{}^{2}\theta \;\cos\;2\varphi \right]$$

where *δ* ^PCS^ is measured as the difference in chemical shift measured in the presence and absence of the paramagnetic metal ion, Δ*χ*_ax_ and Δ*χ*_rh_ are the axial and rhombic components of the Δ*χ* tensor, and *r*, *θ* and *ϕ* are the polar coordinates of the nuclear spin relative to the principal axes of the tensor. PCSs thus encode accurate long‐range structural information that can be used for analysing protein conformations in solution and determining the structures of protein‐protein and protein‐ligand complexes.[^4^, ^5^, ^6^, ^7^, ^8^, ^9^, ^10^, ^11^] PCSs can even be used as the sole experimental restraints for protein fold determination.[^12^, ^13^, ^14^]

Among the paramagnetic lanthanide(III) ions, terbium(III) and thulium(III) stand out for featuring large Δ*χ* tensors and generating PCSs that tend to shift the NMR signals in opposite directions, assisting their assignment relative to the corresponding diamagnetic reference, which can be prepared with a metal of similar ionic radius such as yttrium(III). (Yttrium is not a lanthanide but together with the lanthanides can be referred to as lanthanoid.) These three lanthanoid ions have thus been used extensively to assess the performance of lanthanide tags for PCS measurements in protein NMR.[^5^, ^7^, ^15^] Stable lanthanoid tags enable PCS measurements in living cells and these data can be sufficient for 3D structure determinations of the tagged proteins.[^16^, ^17^]

Gd^III^ is the metal ion with the largest paramagnetic moment, which can be exploited not only for PRE measurements in protein structural biology but, even more prominently, in contrast agents for magnetic resonance imaging.[^18^, ^19^, ^20^] Owing to slow electronic relaxation, Gd^III^ ions have also gained an important role in EPR investigations, as double electron–electron resonance (DEER) experiments deliver precise distances between two Gd^III^ ions ranging between about 2.5 and 8 nm.[^21^, ^22^, ^23^, ^24^] Such measurements deliver unique data for probing the conformation of proteins following the attachment of Gd^III^ tags at two sites.[^25^, ^26^] Unlike traditional nitroxide radicals, the EPR signal of Gd^III^ tags is free of orientation selection effects at high (W‐band) EPR frequencies, enabling sensitive DEER measurements, which can be analysed in a straightforward manner.^[27]^ Furthermore, Gd^III^ tags are insensitive to chemical reduction under physiological conditions, which is important for in‐cell DEER measurements.[^28^, ^29^]

Distance information on the nanometre scale can also be obtained from FRET experiments based on the luminescence of Tb^III^ or Eu^III^ ions. With suitable antennas for efficient photo‐excitation, the luminescence is extremely sensitive and, besides serving applications in structural biology, these lanthanide ions offer outstanding probes in bioassays and for live cell‐imaging.[^30^, ^31^, ^32^, ^33^, ^34^, ^35^, ^36^] In particular, luminescent lanthanide tags are integral to commercial time‐resolved (TR)‐FRET assays to study dynamic processes such as protein conformation, protein–protein interactions and receptor‐ligand binding interactions.[^37^, ^38^, ^39^, ^40^] Ln^III^ complexes of europium and terbium offer advantages over organic fluorophores for FRET‐based assays, including up to millisecond excited state lifetimes, which enables background autofluorescence to be completely removed through time‐resolved measurements, enhancing sensitivity.[^41^, ^42^] In addition, they possess narrow emission bands, which enables selective observation of lanthanide emission, and large pseudo‐Stokes shifts, which minimises self‐absorption processes.[^43^, ^44^]

**Figure 1:**
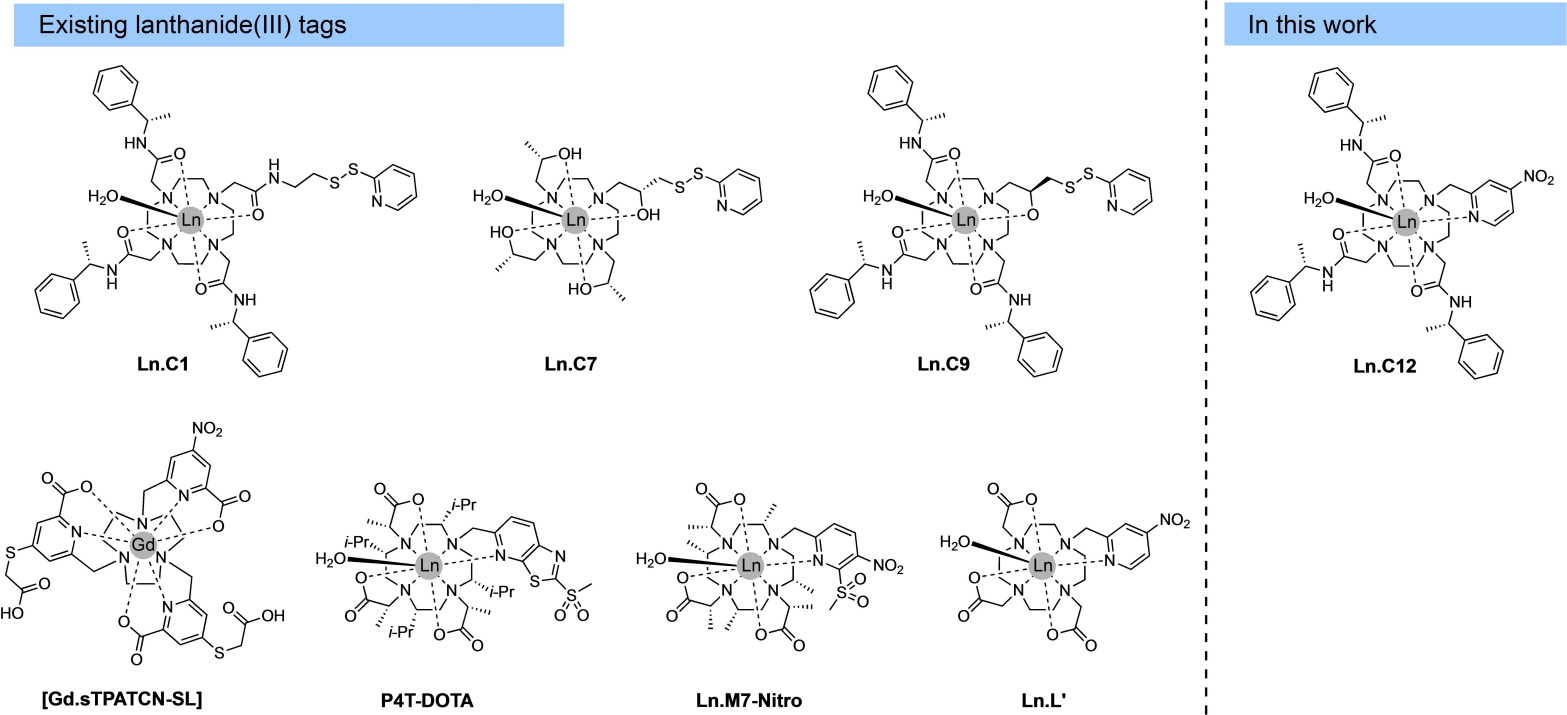
Chemical structures of existing Ln^III^ tags and the new tag **Ln.C12** presented in this work. **Ln.C8** is the same as **Ln.C7**, except for having the opposite chirality in all pendants.

All these applications of lanthanoid ions demand stable attachments to the targeted biological macromolecule, which must be achieved by linking a suitable lanthanide chelating complex to the molecule, as proteins with natural high‐affinity binding sites for lanthanoids are rare.^[45]^ Different approaches for tagging of proteins have been developed over the past two decades, most of which focus on attachment to single cysteine residues, as thiols are chemically more reactive than any other chemical group of the 20 canonical amino acids.[^5^, ^46^] Useful tags for single cysteine residues need to fulfil a number of criteria. i) The tether connecting the lanthanide ion with the sulfur of the cysteine should be rigid, with the smallest possible number of rotatable bonds, as precise structural information can be obtained only if the lanthanide ion does not move relative to the protein.^[47]^ At the same time, the tag must not affect the protein structure. ii) The tagged protein should be chemically stable. Lanthanide tags producing thioether bonds are superior to lanthanide tags attached via disulfide bonds, which are sensitive towards reducing agents. Furthermore, a disulfide bond adds more flexibility to the tether than a thioether group. iii) The lanthanide complex should be convenient to use to minimize protein handling. Most users prefer tags already containing the lanthanide ion over tagging approaches that require titration with lanthanides after installation of the tag, as achieving accurate titration ratios can be difficult. Furthermore, tags containing cysteine‐reactive moieties are preferred over tags that require prior chemical activation of the cysteine residue in the protein. iv) The lanthanide complex of the tag needs to be kinetically and thermodynamically stable in order not to dissociate in aqueous or biological media. v) For measurements of PCSs by NMR spectroscopy, the lanthanide complex must form a single stereoisomer to prevent peak doubling as a consequence of diastereomer formation in the chiral environment of the target protein.[^48^, ^49^, ^50^] vi) Synthesis of the lanthanide tag should be straightforward and affordable. Among the many tags published to date, the P4T‐DOTA and Ln‐M7‐Nitro tags recently published by Häussinger and co‐workers[^51^, ^52^] (Figure 1) fulfil these criteria, except that their synthesis is challenging.

Here, we present a new chiral lanthanide binding tag, **C12**, which is based on a stable cyclen complex, reacts rapidly with cysteine thiols in quantitative yield, produces a thioether bond and a rigid aromatic tether, and is enantiomerically pure and easier to synthesise than the P4T‐DOTA or Ln‐M7‐Nitro tags. The tag combines the reactive *para*‐nitropyridyl group of the previously reported **Ln.L^1^** tag, where the nitro group acts as a leaving group in the reaction with cysteine,^[53]^ with the chiral phenylethylamide pendant arms of the **C1** tag (Figure 1).^[54]^ We demonstrate the performance of this tag with different proteins for measuring PCSs in NMR experiments, Gd^III^−Gd^III^ distances in DEER experiments, and luminescence in peptides and proteins labelled with the Tb^III^ complex. We show that cysteine labelling of the Tb^III^ complex of **C12** elicits a dramatic 100‐fold enhancement in Tb^III^ luminescence from a dark background. The Tb^III^ complex is resistant to oxygen‐mediated quenching and is suitable for use in homogenous time‐resolved luminescence assays, demonstrated by a FRET experiment with a labelled aurora A protein kinase. Finally, we demonstrate fast and complete reaction with selenocysteine, which raises the prospect of site‐selective tagging in the presence of cysteine residues.

## Results

### Lanthanide tag synthesis

**Scheme 1:**
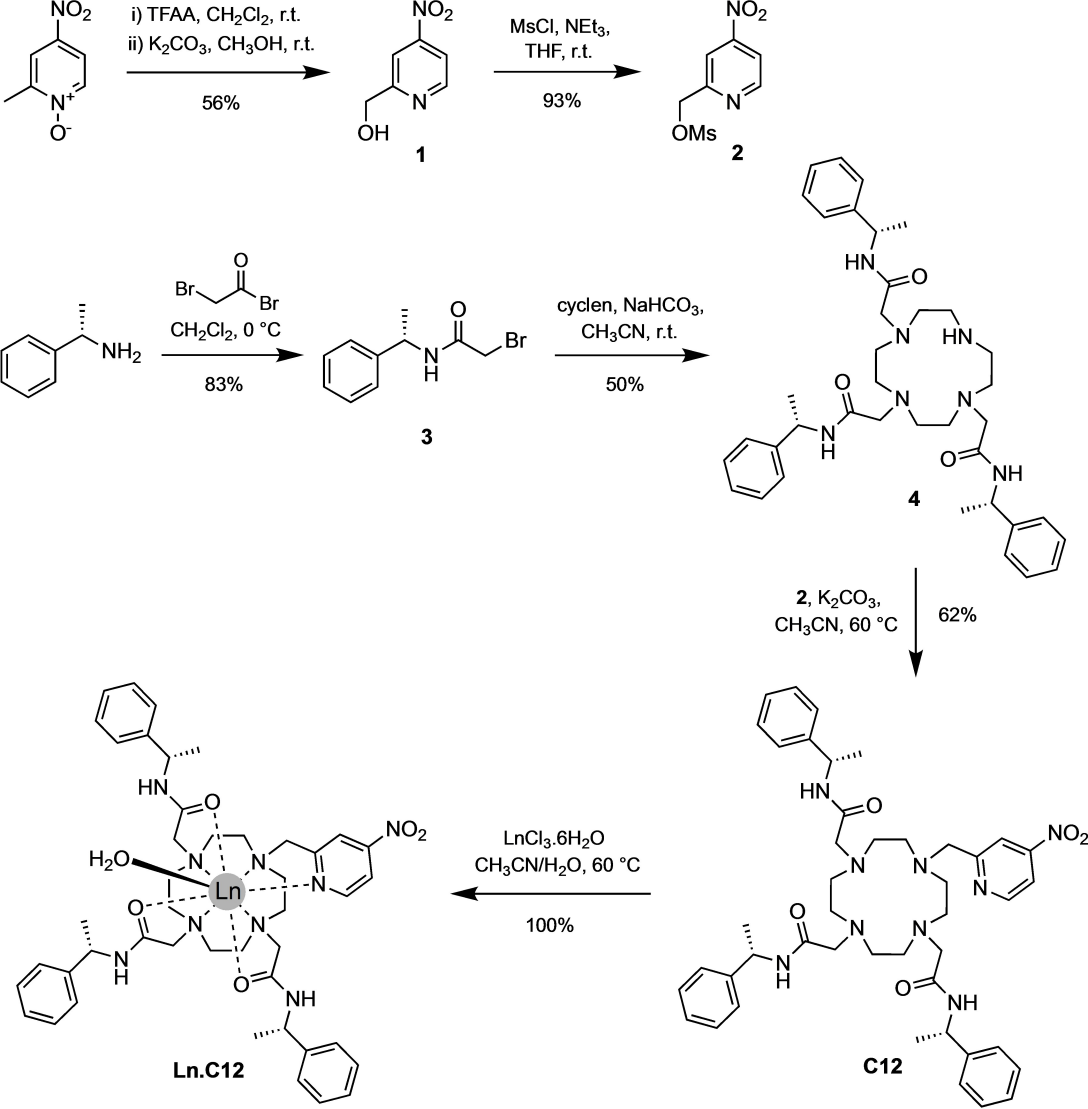
Synthetic scheme for the lanthanide(III) tag **Ln.C12**.

The synthesis of ligand **C12** from 1,4,7,10 tetraazacyclododecane (cyclen) is described in Scheme 1. Briefly, the chiral bromoacetamide arm **3** was prepared by reacting (*S*)‐1‐phenylethanamine and bromoacetyl bromide, followed by *N*‐alkylation onto cyclen to give the macrocyclic compound **4**. Next, *N*‐alkylation of 2‐methyl(sulfonyloxymethyl)‐4‐nitropyridine **2** onto the remaining secondary amine of compound **4** gave the ligand **C12**. Column chromatography was required after each alkylation step to remove impurities; however, the synthesis avoided the need for protecting groups and afforded ligand **C12** in good overall yield. Lanthanoid complexes of **C12** were readily prepared by the addition of one equivalent of the metal chloride salts, LnCl_3_ (Ln=Tb^III^, Eu^III^, Tm^III^, Gd^III^, Y^III^) in a 1 : 1 mixture of acetonitrile/water.

### Photophysical data

**Figure 2:**
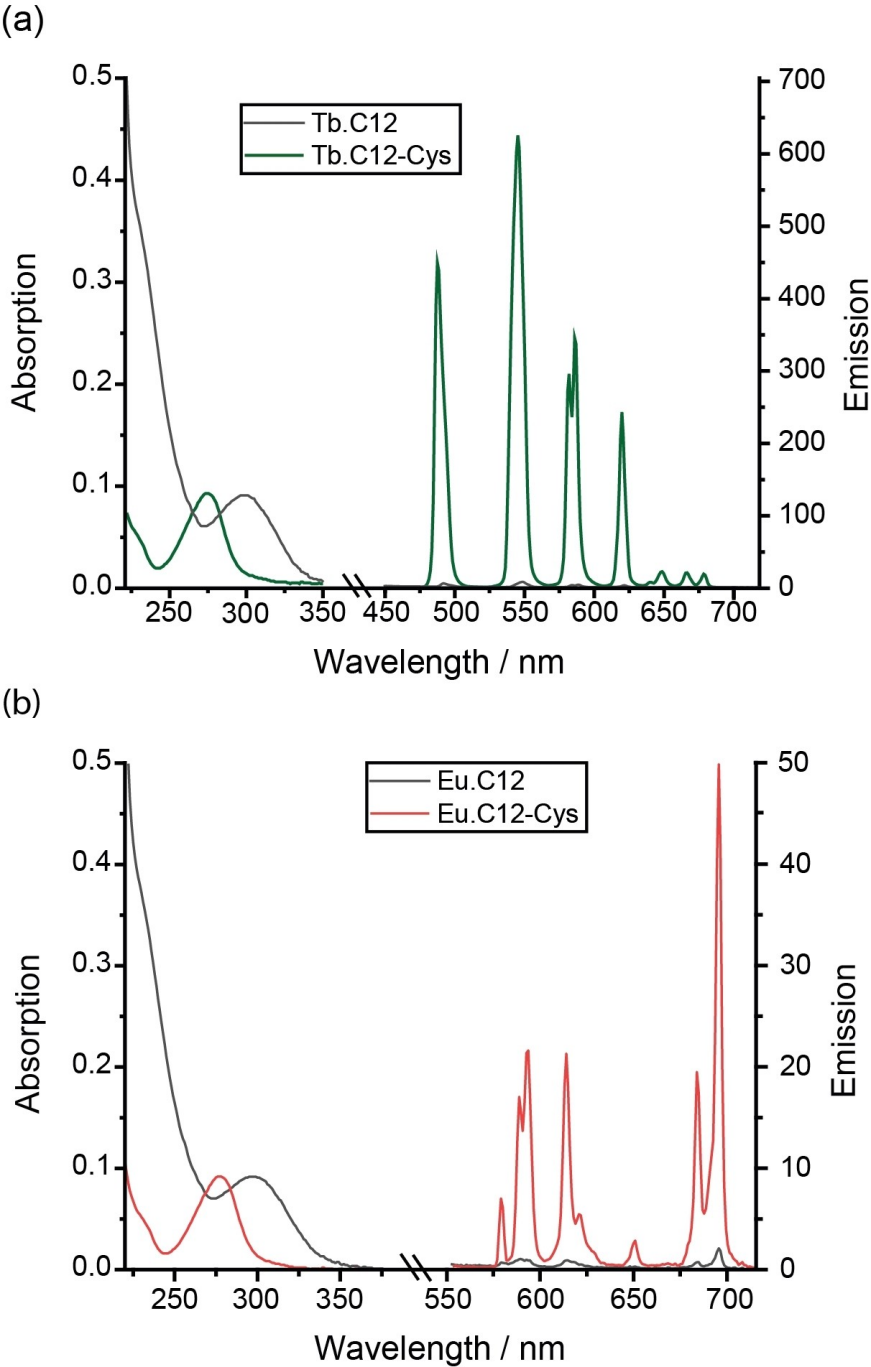
Absorption and emission spectra for a) **Tb.C12** and b) **Eu.C12** and their cysteine derivatives measured in aqueous buffer (10 mM HEPES, pH 7.0). To emphasise the wavelength shift upon reaction with cysteine, the vertical axes of the absorption spectra for the cysteine derivatives of **Tb.C12** and **Eu.C12** were scaled down by factors of 13 and 7, respectively. To confirm that the emission intensity increase was due to the successful reaction of **Tb.C12**, rather than being associated with a noncovalent binding involving displacement of coordinated water, the cysteine derivative was purified by preparative reversed‐phase HPLC.

To establish the fundamental chemical and photophysical properties of **C12**, its performance in luminescence applications was explored first. Photophysical data for the Tb^III^ and Eu^III^ complexes of **C12**, together with their cysteine‐tagged derivatives, are provided in Table 1. The Tb^III^ and Eu^III^ complexes of **C12** have similar absorption spectra (Figure 2), characterized by a broad band centred at approximately 300 nm. Upon excitation of the nitropyridine moiety at 300 nm, the Tb^III^ complex of **C12** displays weak emission with four characteristic bands in the green region (475–630 nm) of the visible spectrum (Figure 2a). The Eu^III^ complex of **C12** emits red light weakly upon excitation at 300 nm, displaying characteristic emission bands in the range 550–720 nm (Figure 2b). The quantum yields of the Ln‐centred luminescence of **C12** were determined by indirect excitation via the nitropyridine antenna to be in the range 0.1–0.2 %. The emission lifetimes for **Tb.C12** were 1.47 ms in H_2_O and 2.50 ms in D_2_O and corresponding values for the Eu^III^ homologue were 0.56 and 2.04 ms. In each case, the change in lifetime in deuterated solvent is consistent with one inner sphere water molecule (*q*=1) for each Ln complex.^[55]^

**Table 1:** Photophysical data for the Tb^III^ and Eu^III^ complexes of **C12** and their cysteine derivatives (10 mM HEPES, pH 7.0).^[a]^

Complex	*λ*_max_ [nm]	*ϵ* [M^−1^ cm^−1^]	*Φ*_em_ [%]	*τ* ${}_{H{}_{2}O}$ [ms]	*τ* ${}_{D{}_{2}O}$ [ms]	*q*
Tb.C12	300	1 200	0.23	1.47	2.50	1.1
Tb.C12‐Cys	278	15 500	20	1.48	2.40	1.0
Eu.C12	297	1 580	0.03	0.56	2.04	1.0
Eu.C12‐Cys	277	11 040	0.90	0.57	2.08	1.0

[a] Values of hydration state *q* (±20 %) were derived using literature methods.^[55]^ Quantum yields were measured using quinine sulfate in 0.05 M H_2_SO_4_ as a standard (*Φ*_em_=52 %).^[56]^ Errors in quantum yields and lifetimes are ±15 %.

### Cysteine tagging reactions

The ability of **Tb.C12** to react with cysteine and other biological thiols was evaluated in water at pH 7.0. Incubation of **Tb.C12** (250 μM) with 4 mM cysteine, homocysteine (hCys) and glutathione (GSH) at 37 °C for 16 h resulted in quantitative reaction of the Tb^III^ complex as indicated by LCMS analysis (Figure S1 in the Supporting Information).

Upon ligation of cysteine, the Tb^III^ complex of **C12** exhibits a remarkable 100‐fold enhancement in Tb^III^‐centred luminescence (475–630 nm; Figure 2a). The Tb^III^ emission is effectively ‘switched on’ upon ligation with cysteine. Similar enhancements in emission intensity were observed upon reaction with homocysteine and glutathione (Figure S2). The absorption spectrum of the purified complex, **Tb.C12**‐Cys, showed a blue‐shifted band centred at 280 nm (Figure 2a), different from the untagged complex **Tb.C12** (*λ*_max_=300 nm). Notably, the extinction coefficient of **Tb.C12**‐Cys was measured to be 15 500 M^−1^ cm^−1^, approximately 13 times higher than for the untagged complex. The overall quantum yield of the Tb^III^ complex increased from 0.2 to 20 % following ligation with cysteine. The emission lifetimes for **Tb.C12**‐Cys in H_2_O and D_2_O were very similar to those obtained for the unreacted complex **Tb.C12** (Table 1), indicating one coordinated water molecule (*q*=1) and confirming that the observed increase in Tb^III^ luminescence was not associated with displacement of a water molecule by cysteine.

The substantial increase in both the quantum yield and the extinction coefficient upon ligation of **Tb.C12** with cysteine means that the overall brightness of the Tb^III^ complex, defined as the product of ϵ and *ϕ*, increases approximately 1100‐fold. In comparison, the Eu^III^ complex of **C12** exhibits a smaller 30‐fold enhancement in Eu^III^ luminescence upon reaction with cysteine (Figure 2b and Table 1) and the overall quantum yield increases only to about 1 %. The ability of **Tb.C12** to switch on its emission from a dark background confers significant advantages over other Ln^III^‐based protein tags that display constant luminescence in their free form, including obviating the need for a washing step to remove the unreacted tag, and providing a luminescence method to report the extent of cysteine tagging under physiological conditions.

**Figure 3:**
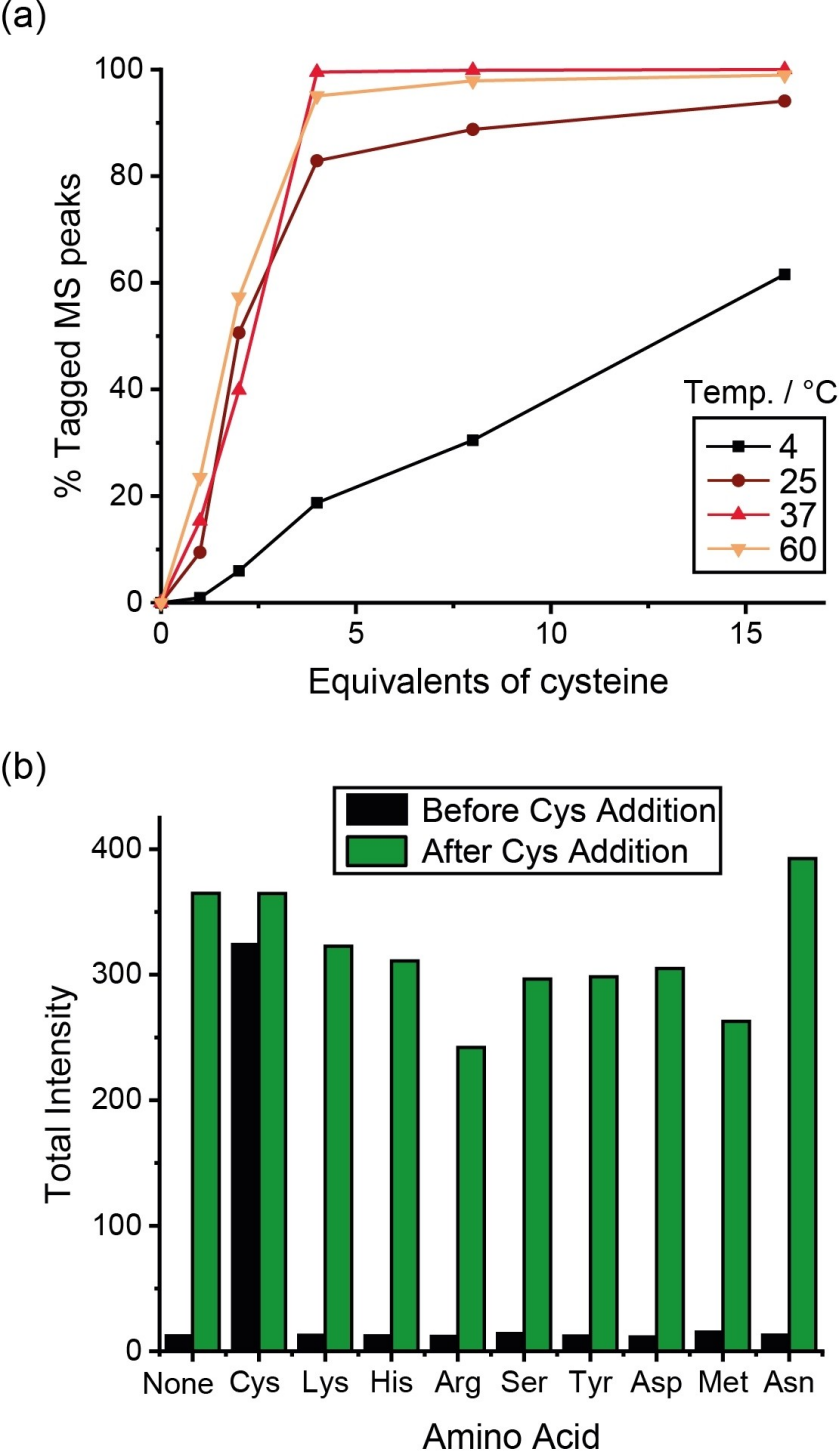
Reaction yields with cysteine. a) Incubation of **Tb.C12** (250 μM) with different equivalents of cysteine in water at pH 7.0 and different temperatures for 16 h. Reaction completion was monitored by ESI mass spectrometry (% cysteine‐tagged MS peak). b) Total terbium emission intensity (*λ*_ex_=280 nm) after 24 h of incubation of **Tb.C12** (250 μM) with various amino acids (4 mM; black), and after a further 24 h of incubation with cysteine (4 mM; green). Incubations were run in water at pH 7.0 and 37 °C. Emission spectra (*λ*_ex_=280 nm) were recorded after tenfold dilution into 10 mM HEPES, pH 7.0.

The optimum number of equivalents of cysteine required to achieve near‐quantitative ligation of **Tb.C12** and **Eu.C12** after 16 h was determined using ESI mass spectrometry, which revealed that four equivalents of cysteine were sufficient to achieve greater than 95 % conversion at 37 °C after 16 h (Figures 3a and S3–S5). Real‐time monitoring of the reaction between **Tb.C12** and cysteine at 37 °C was achieved using UV/Vis and emission spectroscopy, which revealed complete reaction after 3 h (Figure S6).

Next, the chemoselectivity of the tagging reaction was examined by incubation of **Tb.C12** with a range of amino acids (4 mM) containing nucleophilic functional groups in water at pH 7 and 37 °C. After 24 h incubation, no enhancement in Tb^III^ emission intensity was observed with Lys, His, Arg, Ser, Tyr, Asp, Met, Asn or a combination of all of these amino acids, consistent with no reaction occurring (Figure 3b). This was verified by mass spectrometry (Figures S7 and S8), which showed only the signal for unreacted **Tb.C12**. The subsequent addition of 4 mM cysteine resulted in a substantial increase in Tb^III^ emission intensity corresponding to formation of the cysteine complex (confirmed by mass spectrometry, Figures S7 and S8), demonstrating excellent selectivity for cysteine over all other nucleophilic amino acid residues.

Finally, dilution studies conducted with **Tb.C12**‐Cys revealed that nanomolar concentrations of the Tb^III^ tag can be readily detected using standard instrumentation (Figure S9). Further, the **Tb.C12**‐Cys complex is completely insensitive to oxygen‐mediated quenching (Figure S10).

**Tb.C12** was shown to react rapidly and cleanly with the reduced form of GSH but not with the oxidized form (GSSG), which lacks the nucleophilic moiety (similar reactivity of **Tb.C12** was observed with cysteine over cystine, Figure S11).

We reasoned that the selective enhancement in Tb^III^ emission intensity upon reaction with GSH could be used for monitoring the enzymatic conversion of GSSG to GSH. An attractive feature of **Tb.C12** for this purpose is its long luminescence lifetime (1.47 ms in H_2_O, Table 1), which permits time‐resolved analysis of the GSH/GSSG ratio, wherein the introduction of a short delay (60 μs) between the excitation and emission measurement enables complete removal of any short‐lived autofluorescence from biomolecules in the sample, enhancing signal‐to‐noise. Additionally, any light scattering in the instrument is also avoided because the light source is off during acquisition of the emitted light. The first Tb^III^ complex to be reported for monitoring glutathione reductase activity^[57]^ operates by Michael addition of GSH to a pendant maleimide arm, forming a thiol‐maleimide conjugate with enhanced Tb^III^ luminescence. However, maleimide–thiol conjugates are known to be susceptible to thiol exchange and ring opening reactions, which can compromise their longer‐term stability.[^58^, ^59^]

**Figure 4:**
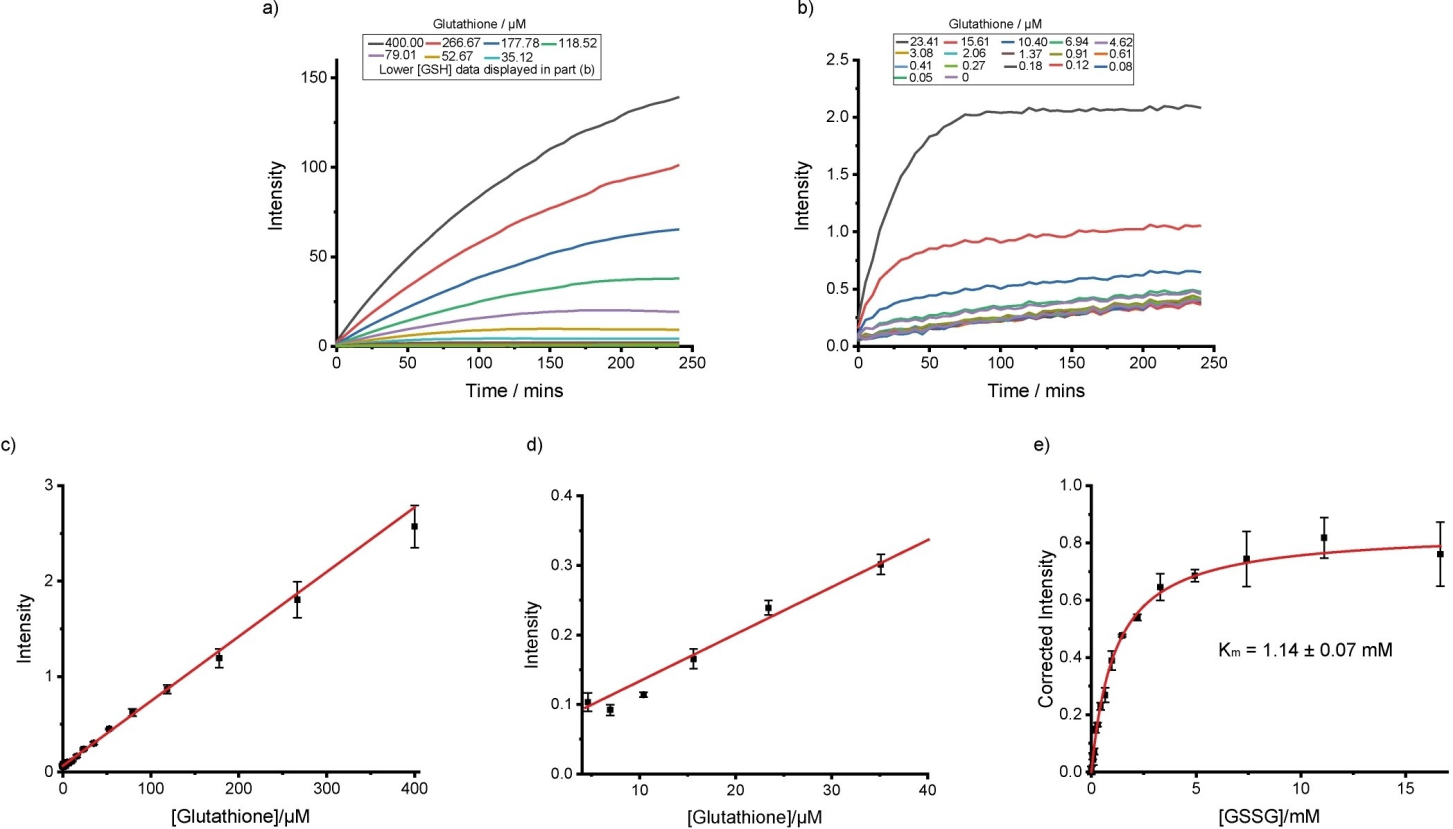
Monitoring glutathione reductase activity by using **Tb.C12**. a) and b) Time‐resolved emission intensity over time following the addition of **Tb.C12** (25 μM) to different concentrations of glutathione in 50 mM Tris**⋅**HCl, pH 7.4. c) and d) Time‐resolved emission intensity (*λ*_ex_=292–366 nm, *λ*_em_=510–500 nm, integration time=60–400 μs) recorded 30 s after addition of **Tb.C12** (25 μM) to different concentrations of GSH in 50 mM Tris**⋅**HCl, pH 7.4. e) Time‐resolved emission intensity (corrected to allow for dilution and background reaction) against initial concentration of oxidised glutathione after adding **Tb.C12** (25 μM) to a glutathione reductase reaction mixture containing NADPH (1 mM), glutathione reductase (0.01 U mL^−1^) and various concentrations of oxidised glutathione (GSSG) in 50 mM Tris**⋅**HCl at pH 7.4 following a 30‐min incubation. Data fitted to the Michaelis‐Menten equation.

**Tb.C12** was added to different concentrations of GSH and the time‐resolved emission intensity recorded as a function of time. The increase in time‐resolved luminescence was immediate (Figure 4a and b) and linearly dependent on GSH concentration even at low μM concentrations and for very short incubation (Figure 4c and d). After a 5‐minute incubation period, the emission intensity increased about threefold. Again, the luminescence of the Tb^III^ complex increased linearly with GSH concentration (Figure S12).

To demonstrate the ability of **Tb.C12** to monitor the reduction of GSSG to GSH, a series of enzyme reactions were carried out using glutathione reductase in the presence of the reducing agent NADPH and different concentrations of oxidised glutathione. After a 30‐minute incubation period, **Tb.C12** (25 μM) was added to the reaction mixture and the time‐resolved emission intensity was recorded. Figure 4e shows a plot of the time‐resolved emission intensity as a function of initial GSSG concentration (corrected to account for the background of an enzyme‐free reaction). As expected, increasing the GSSG concentration increased the emission intensity, consistent with an increased rate of reaction. The data were fitted to the Michaelis‐Menten equation to give a Michaelis‐Menten constant, *K*_m_, of 1.14 mM for GSSG. Thus, the rapid and selective reaction of **Tb.C12** with GSH over GSSG enables the enzymatic reduction of glutathione to be monitored in a convenient, luminescence‐based increase‐in‐signal format.

### Protein ligation reactions for NMR analysis

To explore the performance of **C12** in NMR experiments, we prepared samples of the uniformly ^15^N‐labelled ubiquitin mutant S57C and reacted with the **Tb.C12**, **Tm.C12** or **Y.C12** tags. Quantitative ligation yields were obtained in reactions conducted overnight at room temperature in 20 mM HEPES buffer, pH 7, as indicated by mass spectrometry (Figure S13). Ligation reactions with the proteins IMP‐1N172C, ERp29 S114C and ERp29 G147C similarly resulted in fully tagged protein (Figures S14 and S15). All reactions were performed with 50 μM solutions of protein at a tag/protein molar ratio of 5 : 1.

### Observation of PCSs and Δ*χ* tensors

**Figure 5:**
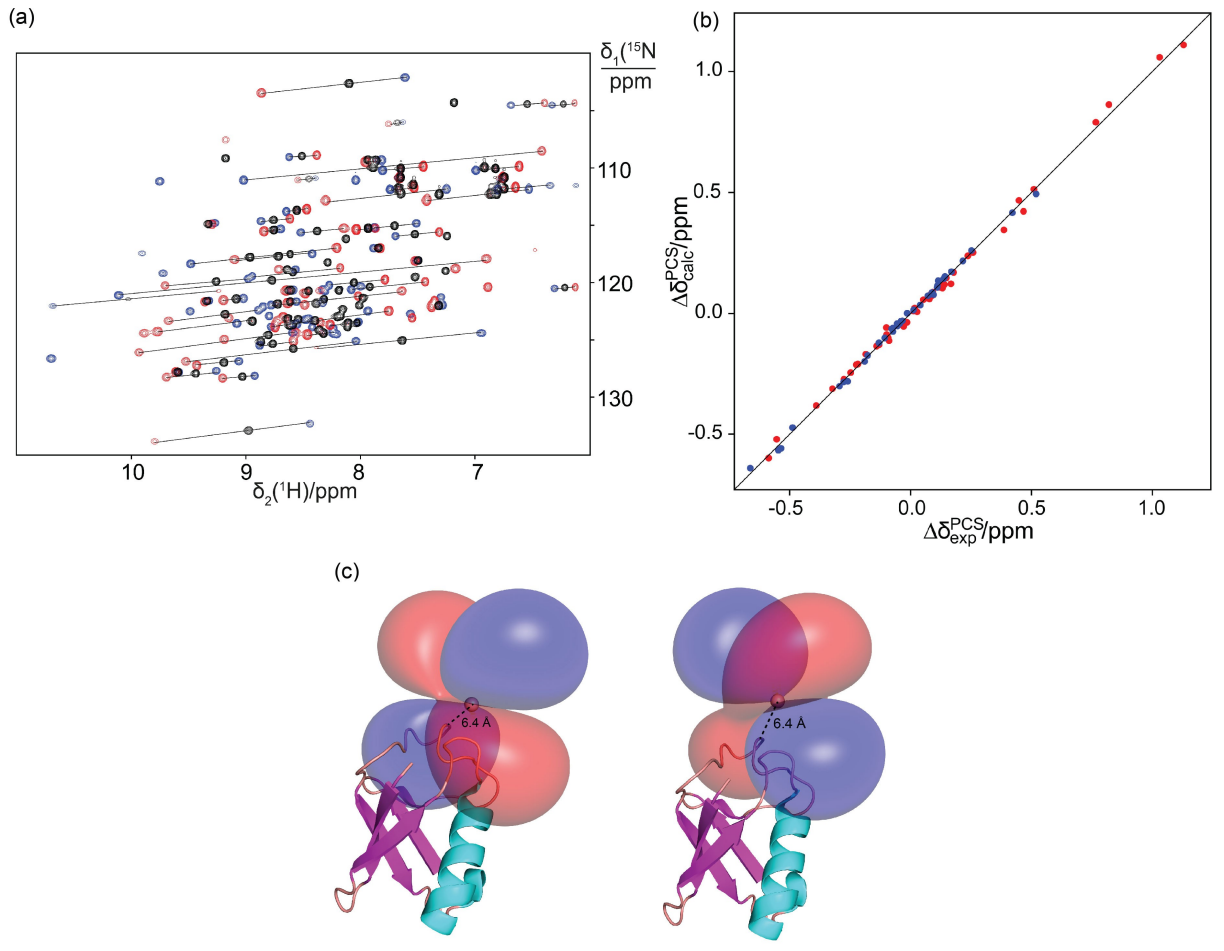
PCSs observed in ubiquitin S57C with **C12** tag. a) Superimposition of ^15^N HSQC spectra. The **C12** tag was loaded with Y^III^ (black), Tb^III^ (red) or Tm^III^ ions (blue). The PCSs of selected amide protons are highlighted by lines connecting the corresponding crosspeaks of the protein with paramagnetic and diamagnetic tags. b) Correlation plot of back‐calculated vs. experimental PCSs for the backbone amide protons of ubiquitin S57C with the **Tb.C12** (red) or **Tm.C12** tag (blue). c) PCS isosurfaces representing the Δ*χ* tensor obtained with the **Tb.C12** (left) or **Tm.C12** (right) tag. The isosurfaces correspond to PCSs of +1 ppm (blue) and −1 ppm (red) and are plotted on the first conformer of the PDB coordinates 2KOX.^[63]^ The backbone of the protein is drawn in a ribbon representation, and the metal position is shown as a sphere. The Δ*χ*‐tensor fit positioned the paramagnetic centre about 6.3 Å from the C^α^ atom of residue 57, as anticipated from the covalent structure of the tag.

NMR experiments of the ubiquitin mutant S57C ligated with the **Y.C12** tag displayed chemical shifts closely similar to the wild‐type protein, indicating little if any structural perturbation introduced by the tag. The **Tb.C12** or **Tm.C12** tags produced significant PCSs (Figure 5a and Table S1). Observation of single peaks for each backbone amide confirmed that the presence of chiral phenylethylamide pendants resulted in a single diastereomer with the protein, which is a prerequisite for avoiding increased complexity of the NMR spectrum, thus enabling straightforward PCS measurements. As the **C12** tag generates fewer rotatable bonds between lanthanide and protein backbone than the **C1** ^[54]^ or **C2** ^[60]^ tags, the **C12** tag is expected to hold the metal ion more rigidly, thus resulting in less averaging between PCSs of different sign. Indeed, the PCSs measured with the **C12** tag tended to be larger than those reported previously with the **Tb.C1** tag (Figure S16)^[61]^ and the quality factor associated with the Δ*χ*‐tensor fit was significantly better (Table 2).

**Table 2:** Δ*χ*‐tensor parameters of ubiquitin S57C tagged with **Tb.C12**, **Tm.C12** or **Tb.C1**.^[a]^

Tag	Δ*χ*_ax_^[b]^ [10^−32^ m^3^]	Δ*χ*_rh_^[b]^ [10^−32^ m^3^]	*x* [Å]	*y* [Å]	*z* [Å]	*α* [°]	*β* [°]	*γ* [°]	*Q* ^[c]^
**Tb.C12**	−13.5(1)	−8.0(1)	13.810	11.561	−3.533	65	56	87	0.01
**Tm.C12**	11.1(2)	3.2(2)	13.810	11.561	−3.533	56	60	67	0.06
**Tb.C1** ^[d]^	−9.06(3)	−2.5(1)	17.220	8.370	−4.000	12	105	151	0.06

[a] The Δ*χ*‐tensor fits used PCSs measured with Tb^III^ and Tm^III^, using Y^III^ as the diamagnetic reference and the NMR ensemble structure of ubiquitin (PDB ID: 2KOX).^[63]^ Fits with the **Tb.C12** and **Tm.C12** tags were to a common set of *x*, *y* and *z* coordinates of the metal position. Euler angles *α*, *β* and *γ* are reported in degrees relative to the structure 2KOX. [b] Uncertainties (in brackets) were determined from fits obtained by randomly omitting 10 % of the PCS data. [c] The quality factor was calculated as the root‐mean‐square deviation between experimental and back‐calculated PCSs divided by the root‐mean‐square of the experimental PCSs. [d] Parameters reproduced from Pearce et al.^[61]^

**Figure 6:**
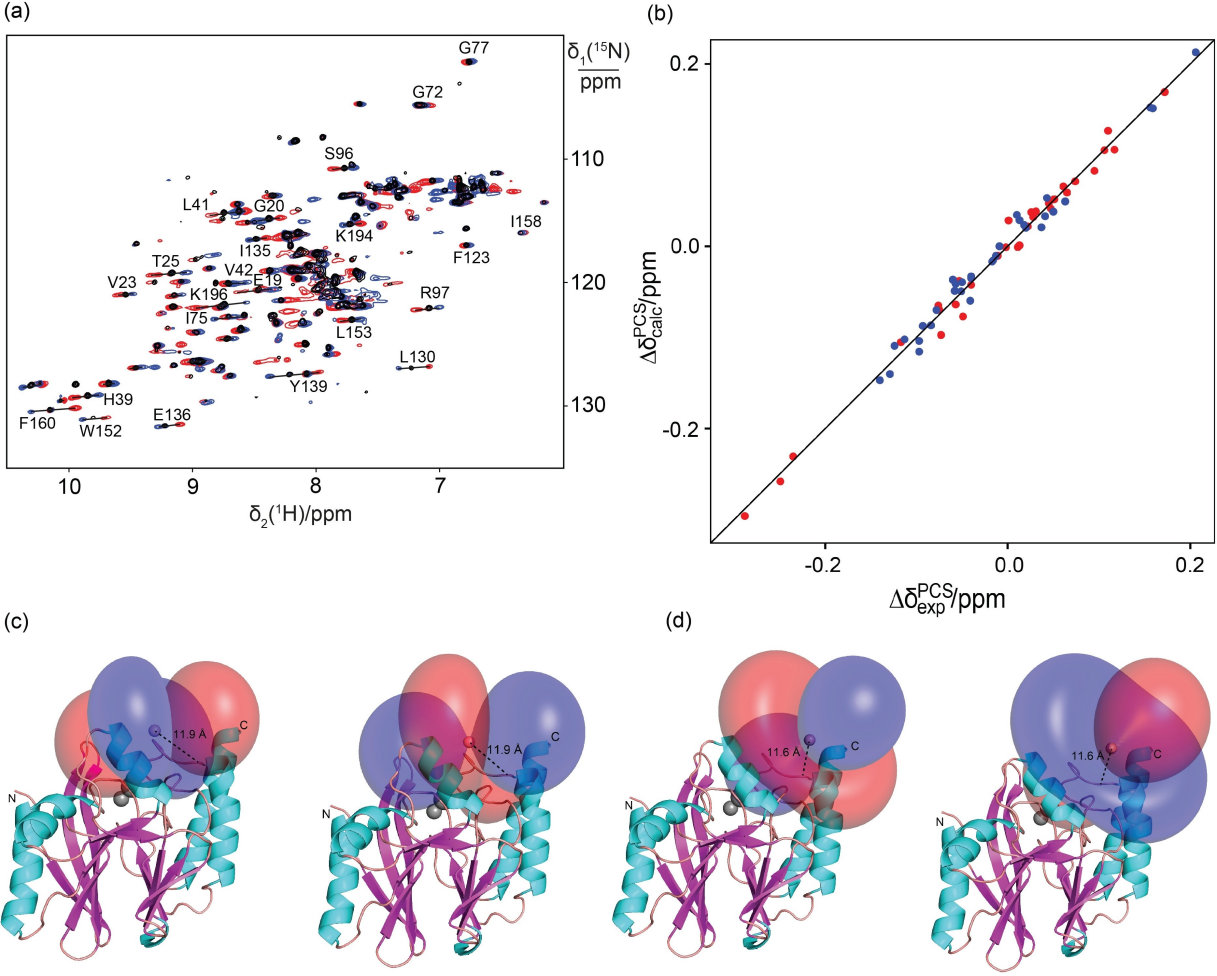
PCSs of the metallo‐β‐lactamase mutant IMP‐1N172C with the **C12** tag. a) Superimposition of ^15^N HSQC spectra of IMP‐1N172C tagged with the **C12** tag loaded with Y^III^ (black), Tb^III^ (red) or Tm^III^ (blue) ions. The PCSs of selected amide protons are identified by lines connecting the corresponding crosspeaks. b) Correlation plot of back‐calculated vs. experimental PCSs for amide protons of IMP‐1N172C tagged with the **Tb.C12** (red) or **Tm.C12** (blue) tag. c) PCS isosurfaces representing the Δ*χ* tensors obtained with the **Tb.C12** (left) or **Tm.C12** (right) tag attached at position 172. The isosurfaces correspond to PCSs of +1 ppm (blue) and −1 ppm (red) and are plotted on the PDB structure 4UAM.^[64]^ The distance of the paramagnetic centre from the C^α^ atom of the tag attachment site is indicated by a dotted line. d) Same as (c), but for the protein with **Tb.C2** (left) and **Tm.C2** (right) tag.

To confirm these results, we also tagged the N172C mutant of the *Pseudomonas aeruginosa* metallo‐β‐lactamase IMP‐1 with **C12** and **C2** tags. The wild‐type protein already contains a cysteine residue, but this residue coordinates the two zinc ions in the active site^[62]^ and proved to be unreactive towards the tags. As in the case of ubiquitin, conservation of the NMR chemical shifts following ligation with **Y.C12** or **Y.C2** indicated maintenance of structural integrity. Following ligation with tags loaded with Y^III^, Tm^III^ or Tb^III^ ions, PCSs were observed both with the **C12** and **C2** tags (Tables S2 and S3). In this case, the **C12** tag produced smaller Δ*χ* tensors than the **C2** tag (Figures 6 and S17), but the *Q* factors of the Δ*χ*‐tensor fits were consistently better (Table 3). In the case of IMP‐1, the Δ*χ* tensor fits identified lanthanoid ion positions that were slightly further from the protein backbone for the **C12** than the **C2** tag (Figure 6c and d). It is unclear whether this indicates de‐coordination of the pyridine moiety in the **C12** tag (despite limited lanthanide hydration indicated by *q*=1 for the derivative with cysteine; see Table 1), or is an artifact of fitting a single Δ*χ* tensor to PCS data generated by a mobile tag, which places the metal ion at variable positions relative to the protein.^[47]^

**Table 3:** Δ*χ*‐tensor parameters of IMP‐1N172C tagged with **C2** and **C12** tags loaded with Tb^III^ or Tm^III^ ions.^[a]^

Tag	Δ*χ*_ax_^[b]^ [10^−32^ m^3^]	Δ*χ*_rh_^[b]^ [10^−32^ m^3^]	*x* [Å]	*y* [Å]	*z* [Å]	*α* [°]	*β* [°]	*γ* [°]	*Q* ^[c]^
**Tb.C12**	13.5(2)	6.4(2)	43.966	85.262	25.852	61	52	112	0.11
**Tm.C12**	−10.5(2)	−5.1(3)	43.966	86.252	25.852	57	54	106	0.12
**Tb.C2**	−18.2(3)	−4.7(2)	40.869	77.901	25.202	22	29	172	0.33
**Tm.C2**	13.9(2)	3.6(3)	40.869	77.901	25.202	20	31	172	0.35

[a] The Δ*χ*‐tensor fits used PCSs measured with Tb^III^ and Tm^III^, using Y^III^ as the diamagnetic reference. The metal coordinates and tensor parameters for the IMP1 mutant are reported relative to the crystal structure of IMP‐1 (PDB ID: 4UAM).^[64]^ [b] Uncertainties (in brackets) were determined from fits obtained by randomly omitting 10 % of the PCS data and the quality factor was calculated as described in footnote [c] of Table 2.

### EPR characterisation of the Gd.C12 tag

**Figure 7:**
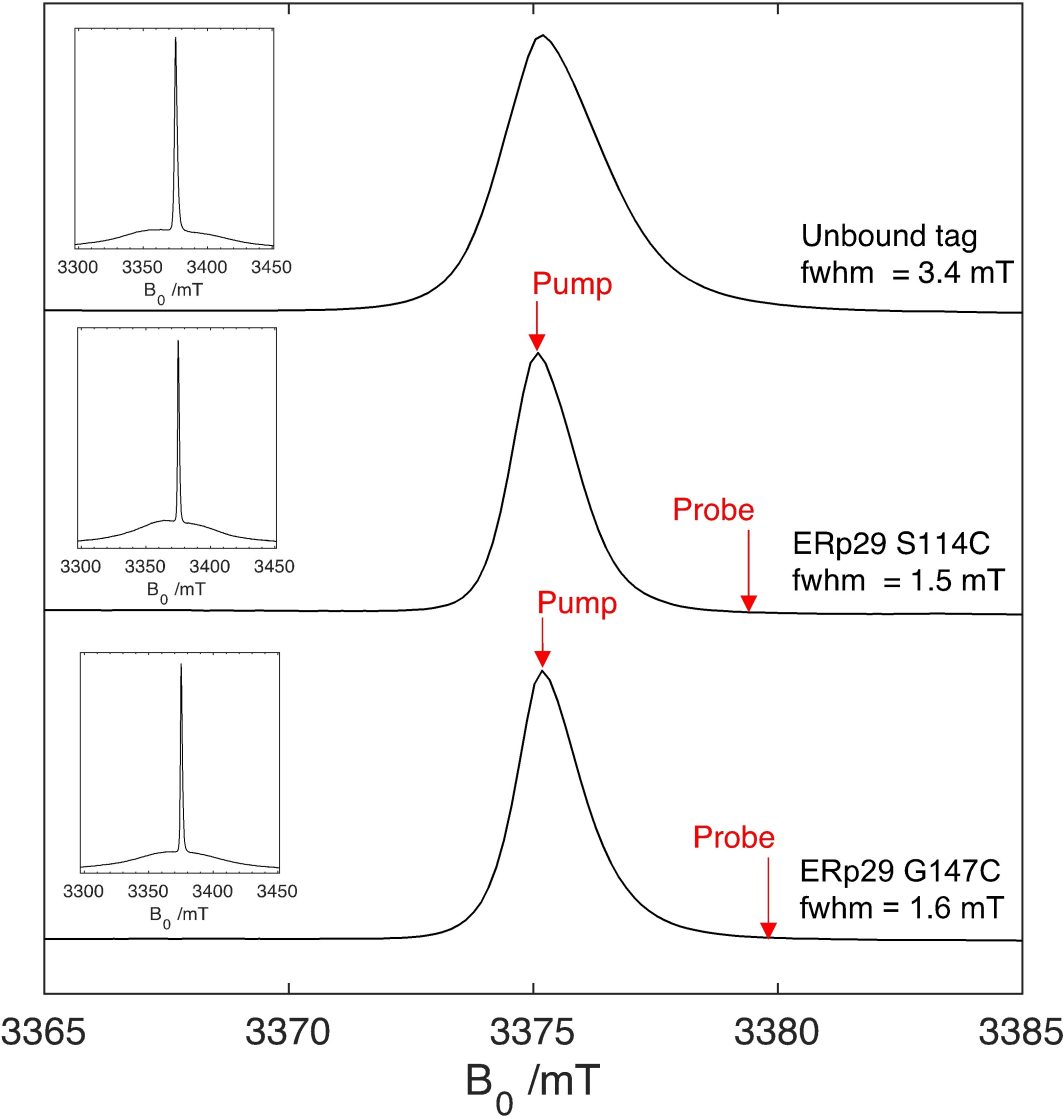
Central transition of echo‐detected EPR spectra of the **Gd.C12** tag, free and bound to cysteine mutants of ERp29 recorded at 94 GHz (scaled to the exact frequency). The full width at half maximum (fwhm) of the central sharp |−1/2 ⟩→|+1/2 ⟩ transition are annotated on each spectrum. The pump and probe pulse positions applied for the DEER experiments of ERp29 S114C and ERp29 G147C are indicated. The inserts show the full spectra. Line‐shape simulations are shown in Figure S20.

To evaluate the performance of **Gd.C12** for DEER applications we turned to ERp29. It is a homodimeric protein and its mutants S114C and G147C have been used previously to assess the performance of different gadolinium tags for distance measurements.[^65^, ^66^, ^67^] Figure 7 compares the EPR line shape of the **Gd.C12** tag ligated to ERp29 S114C and ERp29 G147C with that of the free tag. The line shape of the central m_s_ |−1/2 ⟩→|+1/2 ⟩ transition is similarly narrow as that of the structurally related **C1** and **C9** tags^[66]^ and undergoes minimal change upon attachment of the tag to the protein, except for about two‐fold narrowing of the central transition. *T*_1_ relaxation (120 μs, 10 K) as determined from three‐pulse inversion recovery did not change significantly between the unbound and bound tag (Figure S18, Table S4). Phase memory times *T*_M_ (8–10 μs) as determined from spin‐echo decay experiments were also similar (∼15 % longer, Table S6), provided the protein was incubated in D_2_O over 24 h (Figure S19). As the protein samples were not perdeuterated, the sensitivity of *T*_M_ to hydrogen exchange must be attributed to amide and hydroxy groups, most likely the amides of the pendant arms of the **C12** tag, which are close to the Gd^III^ ion (Figure 1).

### DEER experiments

**Figure 8:**
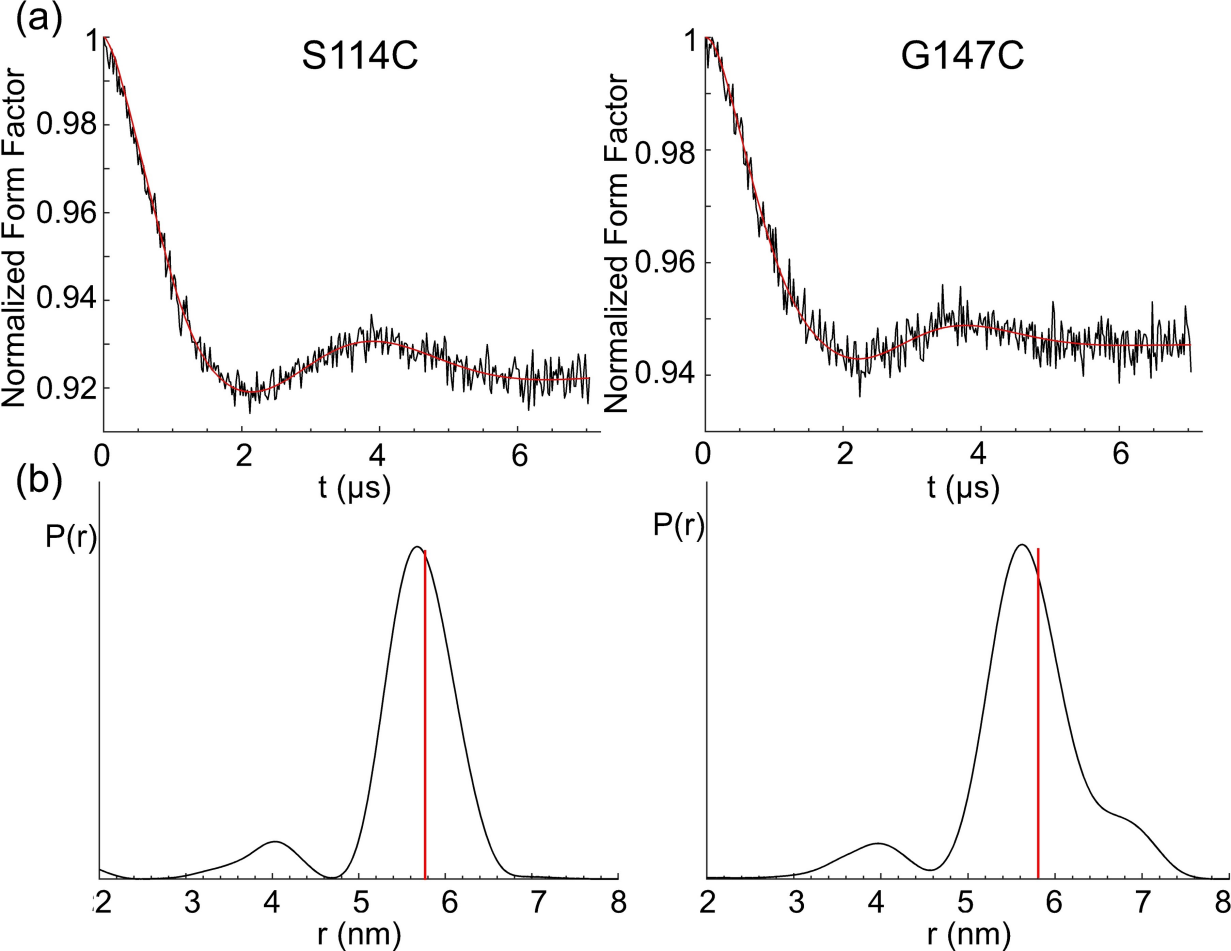
DEER distance measurement of ERp29 S114C (left) and ERp29 G147C (right) tagged with **Gd.C12**. a) Form factor after background subtraction. The vertical axis plots the normalized echo intensity, the red line corresponds to the fitted trace. b) Distance distribution calculated by DeerAnalysis2018.^[70]^ The red lines indicate the maxima of the modelled distance distributions. The full DEER data are shown in Figure S21.

DEER measurements performed with the mutants S114C and G147C of ERp29 ligated with the **Gd.C12** tag are shown in Figure 8. A modulation depth *λ* of about 6–8 % was obtained, which is very good for DEER with Gd^III^.^[68]^ For the mutant S114C and G147C, narrow distance distributions centred at 5.7 and 5.6 nm respectively were observed, in close agreement with modelling by tag rotamer libraries generated with the program PyParaTools^[69]^ (Figure S23). These distances are somewhat shorter than obtained with the **C1** tag, which were 6.1 and 5.7 nm, respectively.^[65]^ The shorter distance obtained with the **C12** tag can be attributed to the shorter linker length to the cysteine residues, which was also reflected in the modelled distance distributions (Figures S22 and S23). Despite a more rigid linker, the experimentally determined distribution widths obtained with **C12** were similar to those obtained with the **C1** tag.

The performance of **C12** is also comparable with the **C7** and **C8** tags, which display distances closer to 6 nm.^[67]^ All three tags have the same number of bonds in the linker between the cyclen ring and cysteine residue. Comparing the distribution widths with those obtained with the **C7** and **C8** tags is more difficult, because these tags yield remarkably narrow distance distributions for ERp29 S114C but, for unknown reasons, unexpectedly broad distributions for ERp29 G147C.^[67]^ As this is not observed for **C12**, this difference must be an artefact, which we speculate the **C12** tag is less susceptible to.

### Time‐resolved FRET using Tb^III^‐labelled aurora A kinase

Distance information on the nanometre scale can also be obtained from FRET experiments utilising luminescent Tb^III^ or Eu^III^ donors. The selective reaction of **Tb.C12** with cysteine‐containing biomolecules, coupled with its bright and long‐lived luminescence signal upon labelling, make the Tb^III^ complex an ideal candidate for use in homogenous time‐resolved FRET assays.[^39^, ^71^, ^72^] Due to the spectral overlap of the emission bands of **Tb.C12** and the absorption spectrum of AlexaFluor 633 (AF633; *λ*_ex_ 633 nm, *λ*_em_ 650 nm), these two molecules were used as a FRET donor and acceptor, respectively. The suitability of thiol‐tagged **Tb.C12** and AF633 as a FRET donor/acceptor pair was established in experiments conducted with GSH‐tagged **Tb.C12** donor and freely diffusible AF633 acceptor, which indicated a rate constant for energy transfer of 1.5×10^9^ M^−1^ s^−1^ (Figure S24).

To demonstrate FRET experiments in a protein, we used a construct of the protein kinase aurora A engineered to contain exactly two surface exposed cysteine residues (D274 N/S278 C/C290 A/H373 C/C393 A mutant). Aurora A regulates entry into mitosis and other processes integral to cell proliferation and is a target of several cancer drug discovery programmes.[^73^, ^74^, ^75^] Incubating the protein with **Tb.C12** at 4 °C and pH 7.4 for 18 h, the maximum enhancement in Tb^III^ emission intensity was observed for a tag/protein molar ratio of 4 : 1 (Figure S25).

**Figure 9:**
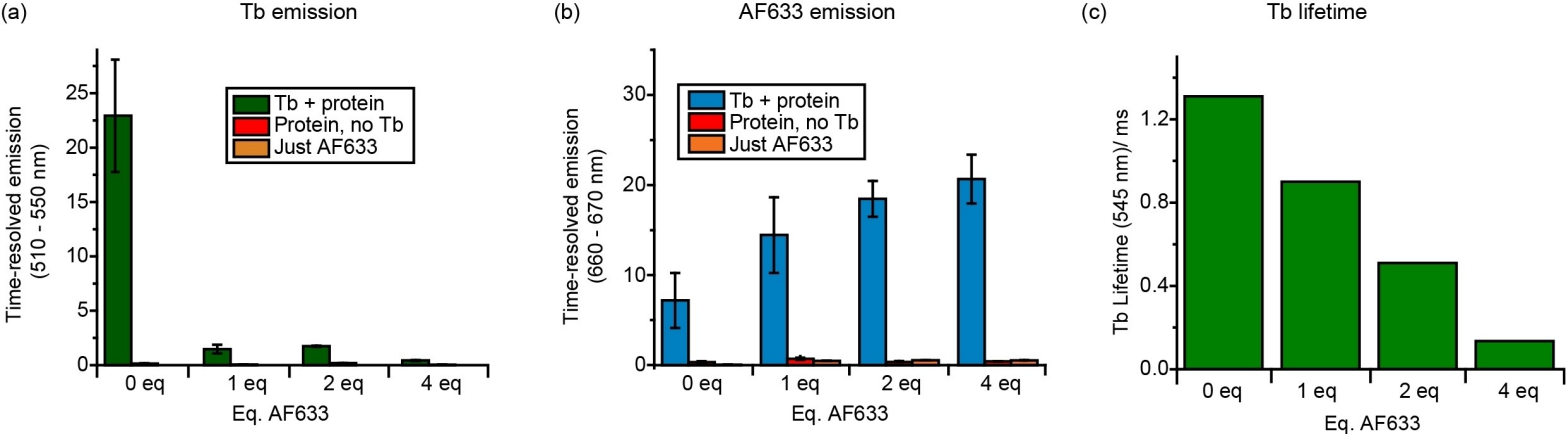
Time‐resolved emission intensity (*λ*_ex_=292–366 nm, integration time=60–400 μs) of Tb^III^ emission (*λ*_em_=510–550 nm) and AF633 emission (*λ*_em_=660–670 nm). The tagging reaction was performed by incubating 15 μM aurora A with 120 mM **Tb.C12** and different amounts of AF633 at 4 °C for 18 h in 50 mM Tris**⋅**HCl, 50 mM NaCl, 1.8 % DMSO at pH 7.4. Control experiments involved aurora A with no Tb^III^ complex (red) or only AF633 (orange). a) Tb^III^ emission after incubation of aurora A with different equivalents of AF633 (relative to aurora A). b) Same as (a), but monitoring AF633 emission. Observation of some background emission without AF633 is expected from the emission spectrum of the Tb^III^ tag (Figure 2a). c) Tb^III^ emission lifetimes (*λ*_ex_=280 nm, *λ*_em_=545 nm) of the samples in (b).

FRET measurements of aurora A were performed following stochastic labelling with the **Tb.C12**/AF633 pair, using 15 μM solutions of aurora A and 120 μM solutions of **Tb.C12**, whilst varying the concentration of AF633 from 0–60 μM. Background fluorescence from the sample was effectively eliminated by applying a time delay of 60 μs between excitation (*λ*_ex_=292–366 nm) and detection (*λ*_em_=510–550 nm for **Tb.C12** or 660–670 nm for AF633). FRET was demonstrated by the presence of AF633 acceptor decreasing the Tb^III^ emission intensity (Figure 9a), time‐resolved emission intensity of the AF633 acceptor, which increased in a concentration dependent manner (Figure 9b), and a concomitant decrease in the Tb^III^ emission lifetime (Figure 9c). These data are consistent with increasing FRET due to an increase in the number of protein molecules stochastically labelled with both donor and acceptor dyes. The labelling sites on Aurora A are expected to be separated by 19–31 Å (no single distance since one site is on a flexible loop) indicating that high FRET signals can be detected in this distance range. Control experiments involving the protein tagged only with the AF633 acceptor confirmed that both the donor and acceptor must be present for FRET to be observed (Figure 9).

We used our measured quantum yield and absorbance/emission spectra to calculate the critical distance (*R*_0_) for the **Tb.C12**‐AF633 FRET pair. We determined this to be 53±4 Å which is similar to that for many other commonly used FRET pairs. We cannot easily calculate the expected FRET efficiency for specific distances using this value of *R*_0_ since our standard calculation assumes free rotation of both dye molecules (i. e., *κ* ^2^=2/3) and our NMR measurements indicate that this is unlikely to be the case for **Tb.C12**. Nevertheless, our measurements of high FRET in the 19–31 Å range are consistent with our calculation which predicts FRET efficiency >78 % for values of *κ* ^2^ for these distances in the physically meaningful range 0.1≤*κ* ^2^≤4. Thus, we conclude that **Tb.C12** is highly suitable for time‐resolved FRET assays.

### Selectivity of the C12 tag for selenocysteine

The broad utility of **C12** for many different types of experiments prompted us to explore its potential for site‐selective tagging of proteins containing selenocysteine residues. The selenol group of selenocysteine (Sec) is much more nucleophilic than the thiol group of cysteine and recently developed technology for genetic encoding of a photocaged selenocysteine residue, in principle, enables site‐specific installation of a selenocysteine residue, although the protein yields obtainable are still too low for routine use.^[76]^ As solvent‐exposed selenocysteine residues are highly prone to forming Se−Se bonds, free selenol groups can be maintained only in the presence of reducing agents,^[77]^ which are incompatible with tags that contain activated disulfide bonds. In the present work, we therefore tested the compatibility of the **C12** tag with reducing agents and its potential for ligation to selenocysteine.

**Figure 10:**
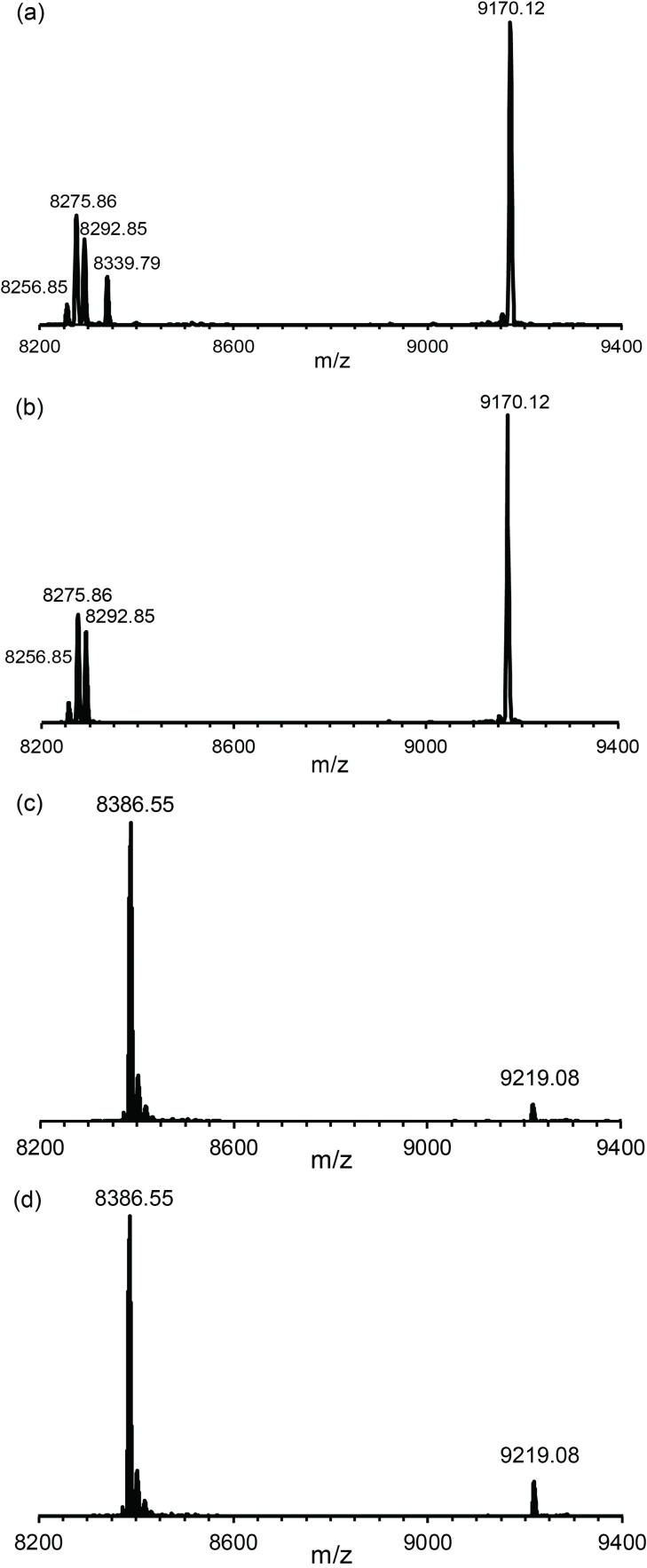
Mass spectra illustrating the much faster reaction of the **Y.C12** tag with selenocysteine than cysteine. a) and b) Reaction with the mutant GB1 Q32Sec after 5 and 10 min, respectively. The calculated masses of the untagged and tagged proteins are 8339.98 and 9172.32 Da, respectively. c) and d) Reaction with the uniformly ^15^N‐labelled mutant GB1 Q32C after 10 min and 5 h, respectively. The masses calculated for 100 % ^15^N‐enriched untagged and tagged proteins are 8388.38 and 9220.73 Da, respectively.

The mutant Q32Sec of the protein GB1 was prepared by cell‐free protein synthesis with the exclusion of cysteine and provision of selenocystine to incorporate selenocysteine in response to the cysteine codon. Tagging reactions with **Y.C12** were conducted in parallel with GB1 Q32Sec and GB1 Q32C. The reaction with GB1 Q32Sec was complete within 10 minutes, whereas only a small fraction of GB1 Q32C had reacted even after 5 h at room temperature (Figure 10). After the tagging reaction, GB1 Q32Sec displayed three additional mass peaks, two of which may be attributed to the reduction of the selenocysteine residue to serine (*m*/*z* 8275.86 Da) and alanine (*m*/*z* 8256.85 Da) caused by the presence of TCEP, which are not amenable to reaction with the **C12** tag. The third minor peak (*m*/*z* 8292.85 Da) may arise from a minor level of cysteine instead of selenocysteine incorporation. These results indicate that the correctly tagged protein was the main species and illustrate the potential of selenocysteine for site‐specific tagging in the presence of free thiol groups.

## Discussion

Many lanthanide tags have been developed and compared for PCS and DEER measurements.[^5^, ^7^, ^24^] Most of the tags are designed for attachment to single cysteine residues in the target proteins, either via a disulfide or a thioether bond. Thioether bonds are generally preferrable, as they produce a shorter tether between metal ion and protein, and disulfide bonds tend to be flexible and readily broken by chemical reduction. Attachment via thioethers has been obtained with tags containing phenylsulfonated pyridines,[^78^, ^79^, ^80^] bromo‐ or iodoacetamides[^81^, ^82^, ^83^] or (methylsulfonyl)thiazolo[5,4‐*b*]pyridines,[^51^, ^78^] or by a thiol‐ene reaction.^[84]^ Phenylsulfonated tags react only slowly with cysteine residues^[78]^ and require high solvent exposure of the thiol group. Halo‐acetamides entail a relatively long and flexible tether with cysteine and tend to be unstable towards lyophilisation.[^81^, ^82^] (Methylsulfonyl)thiazolo[5,4‐*b*]pyridines, such as in the P4T‐DOTA tag (Figure 1), combine high reactivity towards thiol groups with high rigidity of the resulting tether between protein and metal ion, but the synthetic protocol of this group involves a number of steps of modest yield.^[51]^ The methylsulfonyl group on the activated pyridine ring of the Ln‐M7‐Nitro tag (Figure 1) reacts with cysteine thiols within minutes,^[78]^ which may make it difficult to selectively tag selenocysteine in the presence of cysteine.

The 4‐nitropyridyl group of the **C12** tag combines good reactivity with a rigid resulting tether, which is shorter than that obtained with (methylsulfonyl)thiazolo[5,4‐*b*]pyridines. An important advantage of the 4‐nitropyridyl group as the tethering moiety is its ready synthetic accessibility allowing installation on the cyclen ring in just three steps (Scheme 1). In preliminary experiments, we observed that the **C12** tag was significantly more reactive towards cysteine thiol groups than the related **Ln.L^1^** tag (Figure 1),^[53]^ suggesting that the electrophilicity of the pyridine ring is enhanced if the positive charge of the lanthanide ion is not compensated by negatively charged acetate pendants. The effect supports the notion that the metal ion is directly coordinated by the nitrogen of the pyridine ring as designed. Finally, the reactivity of the **C12** tag is compatible with the presence of TCEP to maintain cysteine and selenocysteine in their reduced forms and it is sufficiently stable for shipping at room temperature. Despite its more modest reactivity compared with the **Ln‐**M7‐Nitro tag, we readily obtained 100 % ligation yields with different proteins, without any evidence of reaction with amino acids other than cysteine. Notably, however, the large hydrophobic pendants of the **C12** tag and its positive net charge carry the potential for undesired interactions with the protein. While this cannot be excluded, the present work found no evidence for such effects.

As in the **C1** and **C2** tags,[^54^, ^60^] the chiral phenylethylamide groups on the cyclen ring of the **C12** tag shift the equilibrium between different diastereomeric conformations of the lanthanide complex towards a single species, which is maintained in the adduct with a target protein. As expected for a shorter and more rigid tether between protein and metal ion, the present results confirmed our expectations that the **C12** tag in general delivers larger PCSs and Δ*χ*‐tensor fits of better quality than the **C1** and **C2** tags. The **Gd.C12** tag also proved to be applicable for the measurement of DEER distance distributions, which displayed comparable short distances and distribution widths.

An outstanding feature of the **C12** tag is its capacity to form a stable selenoether bond with selenocysteine. The greater reaction rate of the **C12** tag observed with selenocysteine compared with cysteine is underpinned by the greater nucleophilicity of a selenol versus thiol group near neutral pH. This sets the stage for site‐selective tagging of a protein that contains a single selenocysteine residue, regardless of the presence or absence of cysteine residues. We anticipate that this will present an attractive approach once systems become available that incorporate caged selenocysteine residues with greater yield than hitherto achievable,[^76^, ^85^] as the specificity towards selenocysteine would eliminate the need to mutate native cysteine residues which is a major bottleneck for large proteins that contain several cysteine residues.

Finally, while double‐arm tags attached to two neighbouring cysteine residues immobilise metal ions more easily,[^81^, ^86^, ^87^, ^88^, ^89^, ^90^] suitable sites for double‐cysteine mutations require careful selection and stable attachment of the tag is not guaranteed.^[91]^ Strategies that immobilize the lanthanide ion by simultaneous coordination to two phosphoserine residues^[92]^ or chelating moieties installed on neighbouring cysteine residues[^93^, ^94^] are similarly restricted in the choice of attachment sites. By allowing attachment to a single amino acid residue, the **C12** tag opens a much greater choice of suitable tagging sites.

Among the cyclen tags that react with cysteine by formation of a stable thioether bond,[^17^, ^51^, ^68^, ^78^, ^79^, ^81^, ^82^] the most recent designs use a linker with an aromatic ring that is capable of coordinating the lanthanide ion. This design is attractive as it rigidifies the tether to the protein^[53]^ and in this way limits the averaging between positive and negative PCSs that occurs when the lanthanide complex reorientates relative to the protein. This design is also attractive for DEER measurements in order to obtain the narrowest possible distance distributions. Our present results indicate that the different widths in distance distribution obtained for the sites 114 and 157 in ERp29 mostly reflect the conformational space accessible to the tags, as both **C1** and **C12** yield consistently narrower distribution widths for the site 114 than the site 157 and the latter is more solvent‐exposed. Despite a shorter and more rigid tether in **C12**, however, the widths of the distance distributions were similar, contrary to our simulations which assumed stable coordination between the pyridine nitrogen atom and Gd^III^ ion. In contrast, our NMR data suggested that the **C12** tag restricts translational movements of the lanthanide ion relative to the protein better than the **C1** tag, as manifested by better quality factors obtained in the Δ*χ*‐tensor fits of **Tb.C12** on the cysteine mutants of ubiquitin and IMP‐1 compared to those obtained with the **Tb.C1** and **Tb.C2** tags (Tables 2 and 3).^[47]^ Notably, the **Gd.C7** and **Gd.C8** tags delivered much narrower distance distributions for the ERp29 mutant S114C, but unexpectedly broad distributions for the G147C mutant.^[67]^ A similar discrepancy between these two sites was obtained with the **Gd.C9** tag (Figure 1), indicating that the widths of the distance distributions very much depend on the specific tag and its interactions with the protein environment.^[66]^ This may hold in particular for tags with very rigid linkers, as any chemical tag can potentially also affect the protein structure and rigid tags may be more problematic in this regard. In the case of the **Gd.C12** tag, the distance distribution widths varied less between different sites than for the previously published **Gd.C7** and **Gd.C8** tags.^[67]^ In addition, each backbone amide displayed a single peak in the protein NMR spectra, indicating fast exchange between different tag conformations as far as they occur. More examples will need to be evaluated to confirm the consistency in the performance of the **C12** tag.

On a technical note, the DEER measurements with **Gd.C12** delivered a maximal modulation depth of almost 8 %, which is similar to the record modulation depth of 9 % reported for the [Gd.sTPATCN] spin label.^[68]^ For DEER measurements using nitroxide spin labels, it is well‐known that the phase memory time can be greatly extended by perdeuteration of the protein.[^95^, ^96^] As deuteration of exchangeable hydrogens was sufficient to extend the phase memory time of the **Gd.C12** tag, it may possibly be extended further by synthesizing the tag and/or protein in perdeuterated form. In past experiments of ERp29 S114C tagged with **Gd.C1**, however, perdeuteration did not extend the phase memory time very much.^[65]^

In our luminescence experiments, cysteine labelling of the Tb^III^ complex of **C12** elicited a remarkably large enhancement in both the extinction coefficient (*ϵ* increases from 1200 to 15 500 M^−1^ cm^−1^) and emission quantum yield (*Φ* increases from 0.02 to 20 %). The ability to ‘switch on’ Tb^III^ emission upon labelling is highly advantageous for time‐resolved luminescence assays, as it avoids cumbersome washing/purification steps and permits dynamic imaging of biochemical processes in high‐throughput format. Other protein‐labelling methods which display fluorogenic behaviour, such as SNAP‐tag technology^[97]^ require the attachment of the fluorophore to a quencher group, which is released when the tag reacts with the target biomolecule. In the present work, we exemplified the performance of the **Tb.C12** tag in straightforward microplate reader‐based bioassays, monitoring the enzymatic reduction of glutathione and observing time‐resolved FRET within a model protein kinase, Aurora A. The quantum yield of the thiol‐tagged **Tb.C12** is approximately 10 times larger than that of the europium(III) cryptate (*ϕ*=0.02)^[98]^ used in several commercial homogeneous time‐resolved FRET assays, indicating that a tenfold increase in sensitivity is achievable with the **Tb.C12** tag in such assays. The commercially available Lumi4‐Tb tag, which exhibits unequalled brightness (15 800 M^−1^ cm^−1^ at 340 nm in water vs. 3100 M^−1^ cm^−1^ for **Tb.C12**‐Cys at 280 nm), is not silent in its untagged form.^[33]^

## Conclusion

In summary, the **C12** tag combines many favourable features, including stable lanthanide binding in a complex forming a single conformation, a relatively short tether favouring high‐quality Δ*χ*‐tensor fits, high reactivity towards cysteine and selenocysteine, attachment to a single solvent‐exposed thiol or selenol group, and, last but not least, relative ease of synthesis and convenience of use in the tagging reaction which, important for tagging of selenocysteine, can be conducted in the presence of reducing agents such as TCEP. The Δ*χ* tensors measured by NMR are of useful magnitude, the EPR properties of the **Gd.C12** tag make it suitable for DEER distance measurements and the outstanding luminescent properties of the **Tb.C12** tag open a host of attractive applications in structural biology, enzymology and, potentially, even live‐cell imaging applications. We hope to improve these further by modification of the antenna of the **Tb.C12** tag to increase its excitation wavelength towards the visible region. Overall, we anticipate that the broad applicability and convenience of the **C12** tag will make it an exceptionally popular tool.

## Experimental Section

The protocols for synthesis and characterisation of complexes **Tb.C12**, **Eu.C12**, **Tm.C12**, **Gd.C12** and **Y.C12** are provided in the Supporting Information, as are the protocols for protein production, purification and tagging. The Supporting Information also reports the general procedures for the reaction of **Ln.C12** with low‐molecular‐weight thiols, glutathione reductase reactions and tagging of aurora A with **Tb.C12** and AlexaFluor 633.

**Extinction coefficients and quantum yields**: The extinction coefficients of **Ln.C12** or **Ln.C12**‐Cys (Ln=Tb or Eu; 1 mg mL^−1^) were determined in 10 mM HEPES pH 7.0. Quantum yields were measured using quinine sulfate in 0.05 M H_2_SO_4_ as a standard (*Φ*_em_=0.52, λ_ex_=350 nm).^[56]^

**Luminescence experiments**: Luminescence spectra were recorded on a Camlin Photonics luminescence spectrometer with FluoroSENS version 3.4.7.2024 software. Emission spectra were obtained using a 40 μL Hellma Analytics quartz cuvette (Art no. 111‐10‐K‐40). Excitation light was set at 280 nm (or 300 nm for untagged Ln^III^ complexes), and emission read in the range 400–720 using an integration time of 0.5 seconds, increment of 1.0 nm and excitation slit of 0.2 nm and emission slits of 0.5 nm. Plate reader data were obtained on a BMG Labtech CLARIOstar microplate reader in black Fisherbrand™ 384‐well plates, using a total volume of 40 μL per well.

Emission lifetime measurements were performed on the same instrument. Measurements were taken of 1 mL of 0.1 absorbance samples of Ln complexes in 10 mM HEPES, pH 7.0. Measurements were obtained by indirect excitation of the Ln ion via the pyridine antennae using a short pulse of light at *λ*_max_ (300 nm for untagged complexes, 280 nm for thiol‐tagged complexes), followed by monitoring the integrated intensity of the light emitted at 546 nm (Tb complexes) or 615 nm (Eu complexes), with 500 data points collected over a 10 ms time period. The decay curves were plotted in Origin Labs 2019 version 9.6.0.172, and fitted to the equation:

(1) $$I=A{}_{0}+A{}_{1}\;e^{-kt}$$

where *I* is the intensity at time, *t*, following excitation, *A*_0_ is the intensity when decay has ceased, *A*_1_ is the pre‐exponential factor and *k* is the rate constant for the depopulation of the excited state.

The hydration state, *q*, of the Eu^III^ and Tb^III^ complex of **C12** was determined using the modified Horrocks equation:^[55]^

(2) $$q\left(\mathrm{Eu}\right)=1.2\;\left(1/\tau {}_{H{}_{2}O}-1/\tau {}_{D{}_{2}O}-0.25-0.075\;n\right)$$

(3) $$q\left(\mathrm{Tb}\right)=5\;\left(1/\tau {}_{H{}_{2}O}-1/\tau {}_{D{}_{2}O}-0.06\right)$$

where *τ* ${}_{H{}_{2}O}$ and *τ* ${}_{D{}_{2}O}$ are the emission lifetime times in water and D_2_O, respectively, and *n* is the number of carbonyl‐bound amide NH groups.

**Study of specificity for selenocysteine versus cysteine** : The tagging reactions of the proteins GB1 Q32Sec and GB1 Q32C were carried out in parallel, using 50 μM GB1 mutant, 250 μM **Tb.C12** tag and 1 mM TCEP. Progress of the reaction was monitored by sampling 10 μL aliquots from the reaction mixture after 5, 10, 15, 30, 45 and 60 min, as well as after 5 h. The reaction was quenched by the addition of 0.1 % TFA and snap freezing for storage. The final samples were analysed by intact protein mass spectrometry.

**Intact protein mass spectrometry**: Intact protein analysis was carried out on an Orbitrap Fusion Tribrid mass spectrometer (Thermo Fisher Scientific) connected to a Thermo Fisher Scientific UltiMate 3000 HPLC system equipped with ZORBAX 300SB−C3, 3.5μm, 4.6×50mm HPLC column (Agilent Technologies). Each HPLC run was performed with 500 μL/min linear gradient of solvent A (0.1 % (*v*/*v*) formic acid in water) and solvent B (0.1 % (*v*/*v*) formic acid in acetonitrile), ramping solvent B from 5 % solvent B at the start to 80 % after 12min. Data were collected using an electrospray ionization (ESI) source in positive ion mode. Protein intact mass was determined by deconvolution using the Xtract function in the Qual Browser software tool of the program Xcalibur 3.0.63 (Thermo Fisher Scientific).

**NMR measurements** : All NMR data were acquired at 35 °C on a Bruker 600 MHz Avance NMR spectrometer equipped with a TCI cryoprobe. The PCSs of amide protons were measured in ^15^N HSQC spectra recorded with acquisition times of *t*_1max_=90 ms and *t*_2max_=122 ms. The PCSs were measured in ppm as the chemical shifts in the paramagnetic sample minus the chemical shift in the diamagnetic sample.

**Δ*χ*‐tensor fits**: Δ*χ*‐tensor parameters were determined using the program Paramagpy,^[99]^ using the PCSs of backbone amide protons and atomic coordinates from the Protein Data Bank (PDB ID: 2KOX^[63]^ for the structure of ubiquitin and 4UAM^[64]^ for the structure of IMP‐1). The quality of the fit was assessed by the *Q* factor, which was calculated as the ratio of the root‐mean‐square deviation between experimental and back‐calculated PCSs and the root‐mean‐square of the experimental PCSs.

**EPR characterisation**: All EPR measurements were performed at 10 K on a modified Bruker EPR spectrometer operating at W‐band (94 GHz).^[100]^ The line shape of both the protein‐bound and free **Gd.C12** tag was measured by an electron spin‐echo field‐sweep sequence π/2–*τ*–π–echo, using π/2=40 ns, π=80 ns and *τ*=500 ns. *T*_1_ relaxation was measured by the inversion recovery sequency π–*t*+d*t*–π/2–*τ*–π–echo. The integrated echo intensity was recorded as a function of time *t* incremented in intervals d*t* (1000 ns), using the same pulse lengths as above. *T*_2_ relaxation was measured by recording the decay of the integrated echo intensity with time, using the pulse sequence π/2–*t*+d*t–*π*–t*+2d*t–*echo (d*t*=20 ns).

**DEER measurements**: The standard four‐pulse DEER sequence (π/2(ν_obs_)–*t*_1_–π(ν_obs_)–(*t*_1_+d*t*)–π(Δν_pump_)–(*t*_2_–d*t*)–π(ν_obs_)–*t*_2_–echo) was used with averaging over the initial time delay to remove nuclear modulation artefacts; each scan was acquired by averaging four different *t*_1_ values from 400–562 ns. The DEER echo was observed at 93.94 GHz with π/2 and π pulses of 16 and 32 ns, respectively, and an ELDOR pulse of π=16 ns at 120–130 MHz above the probe frequency. Other parameters used were a repetition rate of 255 μs, d*t*=20 ns and *t*_2_=7.6 μs. The field position for detection was set at the peak of the Gd^III^ spectrum (Figure 7), applying the pump pulse at the centre of the Gd spectrum and the probe pulse on the edge of the m_s_ |−1/2 ⟩→|+1/2 ⟩ transition.

The data were analysed using DeerAnalysis2018^[70]^ and distance distributions were obtained using Tikhonov regularization. The regularization parameter was chosen by the *L*‐curve criterion. Estimation of uncertainties in distance distributions due to background correction were obtained using the validation option in DeerAnalysis.

## Supporting information

As a service to our authors and readers, this journal provides supporting information supplied by the authors. Such materials are peer reviewed and may be re‐organized for online delivery, but are not copy‐edited or typeset. Technical support issues arising from supporting information (other than missing files) should be addressed to the authors.

Supporting Information
