# Supplementary material for: A Chiral Lanthanide Tag for Stable and Rigid Attachment to Single Cysteine Residues in Proteins for NMR, EPR and Time‐Resolved Luminescence Studies
Source: Chemistry. 2021 Jul 29;27(51):13009–23. doi: 10.1002/chem.202101143 (PMC8518945; doi:10.1002/chem.202101143)
Supplement: Supplementary file 1 — Supporting Information [file CHEM-27-13009-s001.pdf]

Chemistry–A European Journal

Supporting Information

A Chiral Lanthanide Tag for Stable and Rigid Attachment to Single Cysteine Residues in Proteins for NMR, EPR and Time-Resolved Luminescence Studies

Iresha D. Herath, Colum Breen, Sarah H. Hewitt, Thomas R. Berki, Ahmad F. Kassir, Charlotte Dodson, Martyna Judd, Shereen Jabar, Nicholas Cox, Gottfried Otting,* and Stephen J. Butler*

Supporting Information

Synthesis and characterisation of ligand C12 and corresponding Ln(III) complexes

Reagents

All chemicals used were purchased from standard chemical suppliers and used without further purification.

High Performance Liquid Chromatography

Preparative RP-HPLC was performed using a Waters 2489 UV/Visible detector performed at 254 nm, a Waters 1525 Binary HPLC pump controlled by the Waters Breeze 2 HPLC system software. Separation was achieved using a semi-preparative XBridge C18 (5 µm OBD 19 × 100 mm) column at a flow rate maintained at 17 mL/min. A solvent system composed of either water (0.05% formic acid) / acetonitrile (0.05% formic acid) or water (25 mM NH_4HCO_3) / acetonitrile was used over the stated linear gradient (usually 0 to 100% organic solvent over 10 min). Analytical RP-HPLC was performed using a XBridge C18 (5 µm 4.6 × 100 mm) column at a flow rate maintained at 2.0 mL/ min using the stated gradient and solvents.

2-(Hydroxymethyl)-4-nitropyridine (1)

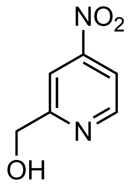

To a solution of 2-methyl-4-nitropyridine-1-oxide (1.00 g, 6.50 mmol) in CH_2Cl_2 (20 mL) was slowly added trifluoroacetic anhydride (1.90 mL, 13.0 mmol) in CH_2Cl_2 (3 mL) and the reaction mixture was stirred at room temperature for 72 hours. The solvent was removed under reduced pressure and the resulting yellow oil was dissolved in methanol (10 mL) and saturated K_2CO_3 solution (5 mL) and stirred at room temperature for 24 hours. The solvent was removed under reduced pressure and the white solid was partitioned between water (30 mL) and ethyl acetate (50 mL) and extracted with ethyl acetate (2 x 50 mL). The combined organic layers were dried over $MgSO_4$, filtered and the solvent was removed under reduced pressure. The alcohol **1** was obtained as a pale yellow solid (560 mg, 56%). No further purification was required. ^{1}H NMR (400 MHz, $CDCl_3$) δ 8.86 (d, *J* = 5.5 Hz, 1H), 8.09 (s, 1H), 7.94 (dd, *J* = 5.5 Hz, 1H), 4.93 (s, 2H), O-H signal not observed. The NMR data are in agreement with those reported previously.[1]

(*S*)-2-Bromo-*N*-(1-phenylethyl)acetamide (3)

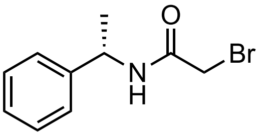

A solution of (*S*)-1-phenylethan-1-amine (6.38 mL, 49.5 mmol) in CH_2Cl_2 (25 mL) was added dropwise to a solution of bromoacetyl bromide (2.16 mL, 27.8 mmol) in CH_2Cl_2 (75 mL) at 0 °C under a nitrogen atmosphere. Following complete addition, the reaction was stirred for 2 hours and allowed to equilibrate to room temperature. Then, the reaction mixture was washed with a 2M hydrochloric acid solution (50 mL) followed by brine (50 mL). The organic layer was dried with $MgSO_4$ and the solvent was removed under reduced pressure to give (*S*)-2-bromo-*N*-(1-phenylethyl)acetamide **3** (5.54 g, 83%) as a white solid. No further purification was required. ^{1}H NMR (500 MHz, $CDCl_3$) δ 7.40–7.25 (m, 5H), 5.08 (d, *J* = 7.0 Hz, 1H), 3.95–3.82 (dd, *J* = 22.9 Hz, *J* = 13.7 Hz, 2H), 1.52 (d, *J* = 7.0 Hz, 3H). ^{13}C NMR (125 MHz, $CDCl_3$) δ: 164.6, 142.4, 128.9, 128.6, 127.7, 126.2, 49.7, 29.4, 21.8. IR(v_{max}/cm^{-1}, neat): 3257, 3060, 2977, 1644, 1543, 1205. HRMS (ESI+): calculated for $[C_{10}H_{12}NOBrNa]^+$ *m/z* 263.9994, found 263.9995.

2,2′,2″-(1,4,7,10-Tetraazacyclododecane-1,4,7-triyl)tris(*N*-((*S*)-1-phenylethyl)acetamide) (4)

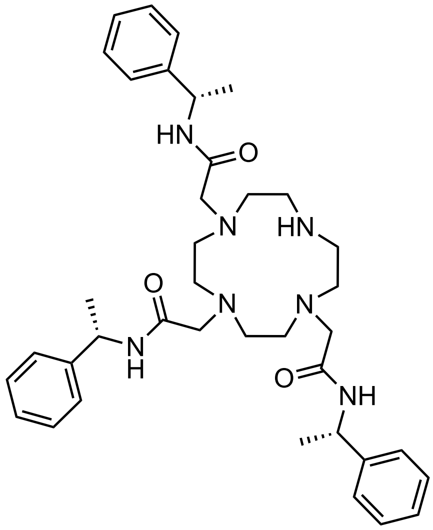

A solution of (*S*)-2-bromo-*N*-(1-phenylethyl)acetamide **3** (0.703 g, 2.90 mmol, 2.5 equiv.) in CH_3CN (10 mL) was added dropwise to a stirred mixture of cyclen (0.20 g, 1.16 mmol) and $NaHCO_3$ (0.243 g, 2.90 mmol) in CH_3CN (50 mL), under a nitrogen atmosphere at room temperature. The reaction mixture was stirred for a further 24 hours, then the solvent was removed under reduced pressure. The residue was dissolved in CH_3Cl (50 mL) and was transferred to a separating funnel. Water (150 mL) was added, and after vigorous shaking the pH was adjusted to 3. The organic layer was isolated and the aqueous phase was washed with CH_3Cl (2 x 50 mL). This procedure was repeated at pH 5, 6 and then 7 to isolate the desired product from over-alkylated byproduct. The organic layers containing the desired product (determined by LCMS analysis) were combined, dried over $MgSO_4$ and the solvent was removed under reduced pressure. The crude material was then purified by column chromatography (silica gel, CH_2Cl_2/NH_3 99.5/0.5, to $CH_2Cl_2/CH_3OH/NH_3$ 88/12/0.5, in 2% increments) to give compound **4** (0.723 g, 50%) as a pale yellow solid. ^1H NMR (400 MHz, $CDCl_3$) δ: 7.50–7.00 (m, 15H), 5.10–4.80 (m, 3H), 3.40–3.00 (m, 6H), 2.70–2.20 (m, 16H), 1.60–1.30 (m, 9H). ^{13}C NMR (100 MHz, $CDCl_3$) δ: 171.7, 171.2, 144.1, 143.9, 128.5, 127.2, 126.7, 126.5, 60.5, 56.9, 55.3, 54.8, 51.9, 50.0, 49.2, 46.5, 22.6, 21.7. IR(ν_{max}/cm^{-1}, neat): 3230, 3030, 2980, 1644, 1534, 1448. R_f (10% CH_3OH/ CH_2Cl_2) = 0.2. HRMS (ESI+): calculated for [M+H$^+$], $[C_{38}H_{54}O_3N_7]^+$ *m/z* 656.4283, found 656.4277.

2,2',2''-(10-((4-Nitropyridin-2-yl)methyl)-1,4,7,10-tetraazacyclododecane-1,4,7-triyl)tris(*N*-((*S*)-1-phenylethyl)acetamide) (C12)

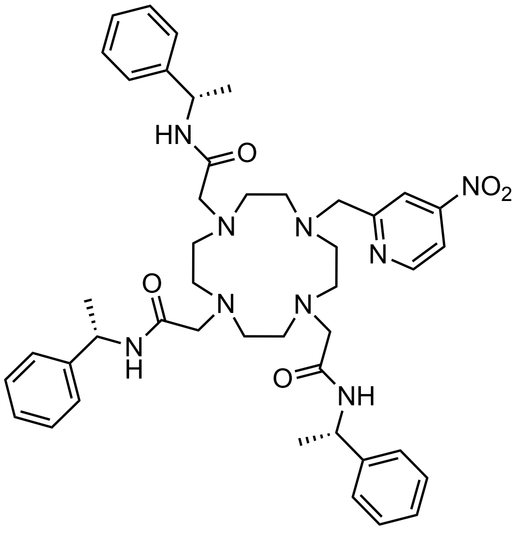

To a solution of alcohol **1** (200 mg, 1.30 mmol) and triethylamine (271 µL, 1.95 mmol) in anhydrous THF (10 mL), was added methanesulfonyl chloride (105 µL, 1.36 mmol) and the yellow reaction mixture was stirred at room temperature for 1 hour. The solvent was removed under reduced pressure and the crude product was partitioned between CH_2Cl_2 (10 mL) and saturated sodium chloride solution (10 mL). The aqueous layer was extracted with CH_2Cl_2 (3 x 10 mL), and the combined organic layers were dried over $MgSO_4$ and the solvent was removed under reduced pressure. The crude mesylate ester **2** was collected as a yellow oil (280 mg, 93%), which was used immediately in the next step.[1]

To a solution of compound **4** (100 mg, 152 µmol) and K_2CO_3 (42 mg, 304 µmol) in anhydrous CH_3CN (5 mL), was added mesylate ester **2** (41 mg, 175 µmol) and the solution was stirred at 60 °C for 24 hours. The orange solution was cooled to room temperature and centrifugated at 120 rpm for 3 minutes. The solution was decanted, and the solid pellet was washed twice with CH_3CN (10 mL). The combined organic layers were concentrated under reduced pressure and the crude material was purified by column chromatography (silica gel; 100% CH_2Cl_2 to CH_2Cl_2/CH_3OH 90/10) to give the desired ligand **C12** as a white solid (75 mg, 62%). ^{1}H NMR (500 MHz, CD_3CN) δ 7.67 (s, 1H), 7.40–6.98 (m, 17H), 5.06–4.48 (m, 3H), 3.46–3.29 (m, 2H), 2.99–1.99 (m, 20H), 1.44–1.21 (m, 9H). ^{13}C NMR (125 MHz, CD_3OH) δ: 172.3, 172.1, 163.4, 155.5, 152.6, 146.7, 146.0, 129.5, 129.3, 128.1, 127.8, 127.3, 127.1, 126.8, 117.2 (m), 115.5, 59.6, 58.1, 53.6-51.2 (m), 50.5, 22.8, 22.3 . IR(ν_{max}/cm^{-1}, neat): 3192, 3055, 2980, 2824, 1653, 1530, 1444, 1354, 1306, 1232, 1098. R_f (10% CH_3OH/ CH_2Cl_2) = 0.2. HRMS (ESI+): calculated for $[M+H^+]$, $[C_{44}H_{57}N_9O_5]^+$ *m/z* 792.4555, found 792.4554.

General procedure for the synthesis of Ln.C12

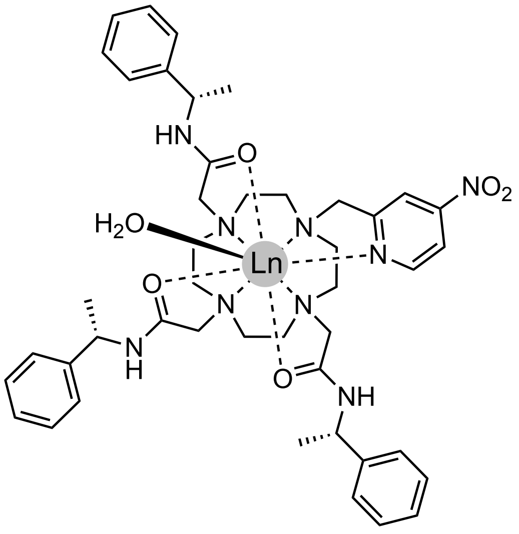

To a solution of ligand **C12** (20 mg, 25.5 µmol) in CH_3CN (2.5 mL) and water (2.5 mL) was added $Ln.Cl_3.xH_2O$ (1.05 equiv.), where Ln = Tb(III), Eu(III), Tm(III), Gd(III), Y(III). The solution was heated to 70 °C for 2 hours. The organic solvent was removed under reduced pressure and the water was removed by freeze drying. The desired complex **Ln.C12** was obtained as a white solid (24 mg, quant.) in each case. Analytical RP-HPLC analysis [XBridge C18 column, gradient: 0 − 100% acetonitrile in 1% v/v formic acid, over 8 min at 0.7 mL per min] revealed a single peak at approximately RT = 3.0 minutes, corresponding to the desired Ln(III) complex (see chromatograms below). Retention times (RT) and mass spectral data for each complex are provided in the table below.

Complex	RT / min	Formula	Calculated m/z	Observed m/z
**Tb.C12**	2.96	$[C_{44}H_{54}N_9O_5Tb]^+$	948.3574	948.3575
**Eu.C12**	3.05	$[C_{44}H_{55}N_9O_5Eu]^+$	942.3533	942.3534
**Tm.C12**	3.02	$[C_{44}H_{55}N_9O_5Tm]^+$	958.3663	958.35
**Gd.C12**	3.02	$[C_{44}H_{55}N_9O_5Gd]^-$	945.3416	945.25
**Y.C12**	2.97	$[C_{44}H_{55}N_9O_5Y]^-$	876.3234	876.30

Reactivity tests of the Ln.C12 tags

General procedure for thiol tagging reactions

Ln.C12 stocks, where Ln = Eu(III) or Tb(III), were made up at 1 mg mL^{-1} in water and the pH adjusted to 7.0. Thiol and amino-acid stocks were generally made to 10 or 100 mM in water and the pH adjusted to 7.0. Thiol stocks were prepared fresh each day. **Ln.C12** (250 μM) and thiol (generally 4 mM or stated concentration) in water, pH 7.0 (or stated buffer), were incubated at 37 °C (or stated other temperature) for 18 hours. The reaction mixture was analysed by LCMS and luminescence emission spectra (following dilution of 5 μL into 45 μL 10 mM HEPES, pH 7.0, and using λ_{exc} = 280 nm, λ_{em} = 400 – 720 nm).

Amino acid selectivity

50 μL of **Tb.C12** (250 μM) and the amino acid(s) (4 mM) in water at pH 7.0 were incubated at 37 °C for 24 hours. 5 μL of the reaction mixture was diluted into 45 μL of 10 mM HEPES at pH 7.0 and emission spectra (λ_{exc} = 280 nm, λ_{em} = 400 – 720 nm) were recorded. In addition, selected reaction mixtures were analysed by LCMS measurements. The selectivity for cysteine was probed in the same way, except that 1.8 μL of 100 mM cysteine (4 mM final concentration) in water at pH 7.0 was added to the reaction mixture.

General procedure for reaction of Ln.C12 with low-molecular weight thiols

4 mL of **Tb.C12** (500 μM) or **Eu.C12** (500 μM) and the thiol compound (8 mM) in water at pH 7.0 were incubated at 37 °C for 18 hours. The reaction mixture was purified by reverse-phase HPLC (0.1% formic acid, 0 – 100% MeCN/H_2O) to give the purified thiol-tagged Ln(III) complexes.

Dilution study with cysteine-tagged Tb.C12

The linearity of the emission intensity (λ_{exc} = 280 nm, λ_{em} = 400 – 720 nm) of pure **Tb.C12**-Cys was probed by a dilution experiment using a 384-well plate in a plate reader to vary the concentration of **Tb.C12**-Cys. The solution was diluted by 60% in each step, starting from a 250 μM solution of **Tb.C12**-Cys in 10 mM HEPES at pH 7.0.

Glutathione titration with Tb.C12

The sensitivity of the emission response of **Tb.C12** towards glutathione was evaluated by a titration using a 2/3 dilution regime in a 384-well plate to vary the concentration of glutathione.

Experiments were conducted in triplicate as follows. 15 μL of 50 mM Tris-HCl at pH 7.4 was placed into all wells, except for the first well, which contained 45 μL of glutathione (800 μM). 30 μL was transferred from the first well to the second well, and mixed. This was repeated until the second last well, where the 30 μL was discarded and the last well left without glutathione. Immediately prior to reading, 15 μL of **Tb.C12** (50 μM) was added to each well and the time-resolved emission intensity recorded every 5 minutes (λ_{exc} = 292 – 366 nm, λ_{em} = 520 – 560 nm, integration time = 60 – 400 μs).

Glutathione reductase reaction

A titration using a 2/3 dilution regime in a 384-well plate was used to vary the concentration of glutathione. Experiments were performed in triplicate, including the controls without enzyme, as follows. 20 μL of 50 mM Tris-HCl at pH 7.4 was placed into all wells, except the first well. 60 μL of oxidised glutathione (25 mM) was placed into the first well. 40 μL was transferred from the first well to the second well, and mixed. This was repeated until the second last well, where the 40 μL was discarded and the last well left without oxidised glutathione. 10 μL of a solution containing glutathione reductase (0.03 or 0 U mL^{-1}) and NADPH (3 mM) was added to each well to start the enzyme reaction. The plate was incubated for 30 minutes, after which 10 μL of **Tb.C12** (100 μM) was added to each well and the plate incubated for 5 minutes. The time-resolved emission intensity was recorded (λ_{exc} = 292 – 366 nm, λ_{em} = 520 – 560 nm, integration time = 60 – 400 μs).

The mean of time-resolved emission intensities was calculated, and the no-enzyme control values subtracted from the values with enzyme. The value obtained was multiplied by 4/6 (correction due to volume change and formation of 2 GSH from 1 GSSG) and this was plotted against initial concentration of GSSG, with errors representing the standard errors in the mean. This was fitted to a Michaelis-Menten equation using OriginLab 2019.

Production, purification and tagging of proteins

Plasmid constructs

The ubiquitin S57C construct was designed with a C-terminal Ser-His_6 tag, ERp29 S114C and ERp29 G147C with C-terminal His_6 tag and GB1 with an N-terminal T7 gene 5 tag (coding for MASMTG) and a C-terminal TEV protease cleavage site followed by a His_6 tag. The respective DNA constructs were cloned into the *Nde*I and

*Eco*RI sites of the pET-3a plasmid.[2] The gene of IMP-1 N172C was cloned between the *Nde*I and *Xho*I restriction sites of the pET-47b(+) plasmid.[3] In order to remove the natural cysteine residue of ERp29, the ERp29 mutants also contained the mutation C157S.

Protein production

All isotope-labelled protein samples were expressed in *E. coli* BL21(DE3) cells.[3] To produce uniformly ^{15}N-labelled ubiquitin S57C, the cells were grown at 37 °C in Luria–Bertani (LB) medium containing 100 μgL^{-1} ampicillin until the OD_{600} reached 0.6–0.8 and were then transferred to 300 mL of M9 medium (6 gL^{-1} Na_2HPO_4, 3 gL^{-1} KH_2PO_4, 0.5 gL^{-1} NaCl) supplemented with 1 gL^{-1} of $^{15}NH_4Cl$. Following induction with isopropyl-β-D-thiogalactopyranoside (IPTG, final concentration 1 mM), the cells were incubated at room temperature for 16 hours. In the case of uniformly ^{15}N-labelled IMP-1 N172C, 50 μgL^{-1} kanamycin was used in place of ampicillin.

Unlabelled ERp29 S114C was produced in *E. coli* BL21(DE3) in 1 L LB medium containing 100 μgL^{-1} ampicillin. Cells were grown at 37 °C until the OD_{600} reached 0.6–0.8. At this point expression was induced with IPTG and the cells kept at room temperature for 16 hours.

The GB1 mutants Q32C and Q32Sec were produced by cell-free protein synthesis (CFPS) following a previously described protocol.[4] Briefly, continuous exchange CFPS[5, 6] was conducted at 30 °C for 16 h, using 1 mL inner reaction mixture and 10 mL outer buffer. The mutant proteins were produced from the PCR-amplified DNA produced with mutation primers and eight-nucleotide single-stranded overhangs to allow cyclization by ligase present in the CFPS reaction.[7] The GB1 Q32C mutant was produced using ^{15}N-labelled amino acids. GB1 Q32Sec was produced by omitting cysteine and adding 1 mM L-selenocystine at a final concentration of 1 mM. The construct was the same as that published by Welegedara et al. (reference 4) with N- and C-terminal expression tags (full amino acid sequence: MASMTGMTYKLILNGKTLKGETTTEAVDAATAEKVFKCYANDNGVDGEWT YDDATKTFTVTEENLYFQGHHHHHH). Selenocystine was reduced to selenocysteine by the presence of 10 mM dithiothreitol (DTT).

Expression of His-tagged Aurora-A kinase in *E. coli* BL21(DE3) cells and purification using IMAC and size-exclusion chromatographies was carried out as previously described.[8, 9]

Protein purification

Ubiquitin S57C was purified following a protocol published earlier[10] for purification of His_6tagged proteins. After purification, the protein was desalted using a HiPrep Desalting 26/10 column (Cytiva, USA) equilibrated with buffer A (50 mM Tris-HCl, pH 8.0, 300 mM sodium chloride, 1 mM DTT) and the His_6 tag was removed by digestion with ubiquitinase[7] for 4 hours at 37 ^{o}C.

The same protocol was followed for purification of unlabelled ERp29 except that the His_6 tag was not cleaved off. To purify uniformly ^{15}N-labelled IMP-1, the protocol was modified by resuspending the cells in buffer B (50 mM HEPES, pH 7.5, 100 μM $ZnSO_4$) for lysis by the Avestin Emulsiflex C5. The supernatant of the centrifuged cell lysate was loaded onto a 5 mL SP column, the column was washed with 20 column volumes buffer C (same as buffer B but with 50 mM NaCl) and then the protein was eluted with a gradient of buffer D (same as buffer B but with 1 M NaCl).

The GB1 mutants expressed by CFPS were purified using a 1 mL His GraviTrap column (GE Healthcare Life Sciences, USA) equilibrated with buffer E (50 mM Tris-HCl, pH 7.5, 300 mM NaCl, 5% glycerol, 1 mM DTT), washed with buffer F (same as buffer A but with 20 mM imidazole) and eluted with buffer G (same as buffer A but with 500 mM imidazole).

Tagging of cysteine mutants with C12 tags

To ensure that all cysteine residues were reduced, DTT was added to a 0.1 mM solution of the protein to a final concentration of 4 mM. After incubation for 1 hour, excess DTT was removed using an Amicon ultracentrifugation tube (MWCO 3 kDa) and the buffer exchanged 20 mM HEPES, pH 7.0. The reduced protein was added slowly into 5 equivalents of the **C12** tag loaded with the requisite lanthanide ions and dissolved in tagging buffer. The reaction mixtures were left at room temperature overnight with shaking. Completion of the ligation reaction was confirmed by mass spectrometry. The tagged proteins were buffer exchanged either to 20 mM phosphate, pH 6.5, for NMR measurements or to 20 mM MES in D_2O, 100 mM NaCl, pH 4.9 (uncorrected pH meter reading), for EPR experiments. Perdeuterated glycerol was added to reach a 20% (v/v) final composition for EPR samples.

Aurora A tagging with Tb.C12

All measurements were carried out using the D274N/S278C/C290A/H373C/C393A mutant of Aurora A (residues 122 – 403). This mutant incorporates the D274N (catalytically inactivating) mutation[11] on a pseudo-wildtype construct of Aurora A in which all surface cysteines have been mutated to alanine[12]. S278C and H373C mutations were incorporated for site-specific dye labelling.

Aurora A (D274N/S278C/C290A/H373C/C393A mutant) was passed through a desalting column in buffer E (50 mM Tris-HCl, pH 7.4, 50 mM NaCl) and the concentration of the eluant calculated by UV/vis absorption. 20 μL of the protein (15 μM) and **Tb.C12** (0, 15, 30, 60 or 120 μM) in buffer E with 1.2% DMSO were placed into a 384-well plate in triplicate. The well plate was incubated at 4 °C for 18 hours. The wells were then diluted with 20 μL of buffer E. The well plate was read by time-resolved emission (λ_{exc} = 292 – 366 nm, λ_{em} = 515 – 565 nm, integration time: 60 – 400 μs). The triplicate reaction mixtures were combined and emission spectra (λ_{exc} = 280 nm) and luminescence lifetime (λ_{exc} = 280 nm, λ_{em} = 545 nm) were measured.

Aurora A reaction with Tb.C12 and AlexaFluor 633

DiCys Aurora A (D274N/S278C/C290A/H373C/C393A) was passed through a desalting column in buffer E, and the concentration of the eluant calculated by UV/vis absorption. 20 μL of the protein (15 or 0 μM), **Tb.C12** (120 or 0 μM) and Alexafluor 633 (AF633) (0, 15, 30 or 60 μM) in buffer E with 1.8% DMSO were placed into a 384-well plate in triplicate. The well plate was incubated at 4 °C for 18 hours. The wells were then diluted with 20 μL of buffer E. The well plate was read by time-resolved emission (λ_{exc} = 292 – 366 nm, λ_{em} = 515 – 565 nm, integration time: 60 – 400 μs), and the mean and standard error in the mean calculated. The triplicate reaction mixtures were combined and emission spectra (λ_{exc} = 280 nm) and selected luminescence lifetimes (λ_{exc} = 280 nm, λ_{em} = 545 and 650 nm) were measured.

Calculation of the critical FRET distance R_0

R_0 was estimated using

$$R_0 = 0.211\ (\kappa^2\, J(\lambda)\ \eta^{-4}\ \Phi_D)^{1/6} \qquad (1)$$

where λ is the wavelength, the orientation factor κ^2 was set to 2/3, the refractive index η was set to 1.333, the quantum yield of the donor Φ_D was set to the value found for **Tb.C12** conjugated to cysteine (Table 1 in the main text), and the overlap integral $J(\lambda)$ was calculated using the fluorescence (F_D) and absorbance (ε_A) spectra in the equation

$$J(\lambda) = \int F_D(\lambda)\ \varepsilon_A(\lambda)\ \lambda^4\ d\lambda \qquad (2)$$

The measured absorbance spectrum was converted into extinction coefficients using ε_A(632 nm) = 239,000 $cm^{-1}M^{-1}$. The uncertainty in R_0 was estimated by propagating values of ±10% for the experimental values of $J(\lambda)$ and the refractive index, and ±15% for F_D.

FRET efficiency (E) was calculated using

$$E = \frac{R_0^6}{r^6 + R_0^6} \qquad (3)$$

where r is the distance between the two fluorophores and R_0 is the critical distance for the dye pair calculated in equation (1).

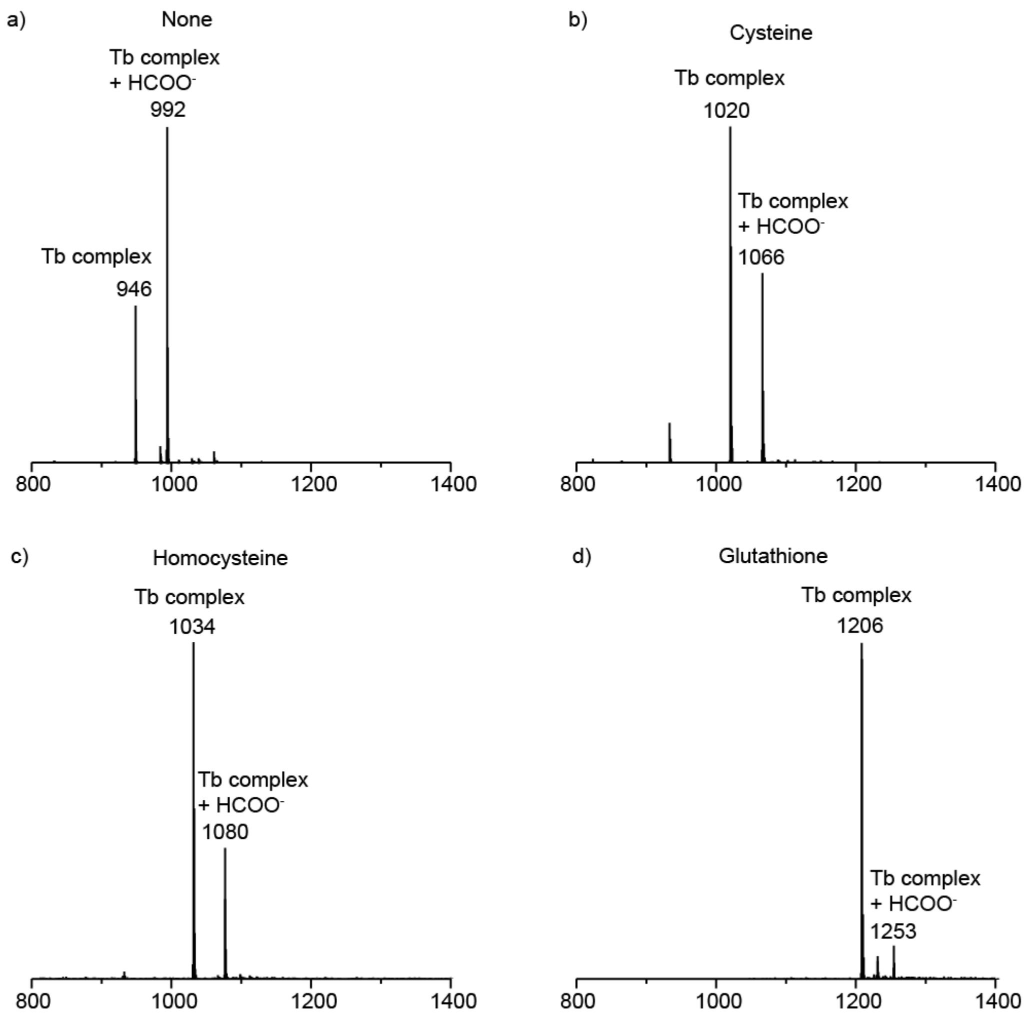

Figure S1. Mass spectra (ESI, negative mode) after 16 hour incubation of **Tb.C12** (250 μM) with 4 different thiols (4 mM) in water, pH 7.0 at 37°C. (a) Without thiol compound. (b) With cysteine. (c) With homocysteine. (d) With glutathione. Quantitative reaction is indicated by the complete loss of the signal for **Tb.C12** (m/z = 946). The major peaks with m/z = 1020, 1034 and 1206 correspond to the single charged thiol-tagged complexes [**Tb.C12**-Cys – 4H]$^-$, [**Tb.C12**-hCys – 4H]$^-$ and [**Tb.C12**-GSH – 4H]$^-$, respectively.

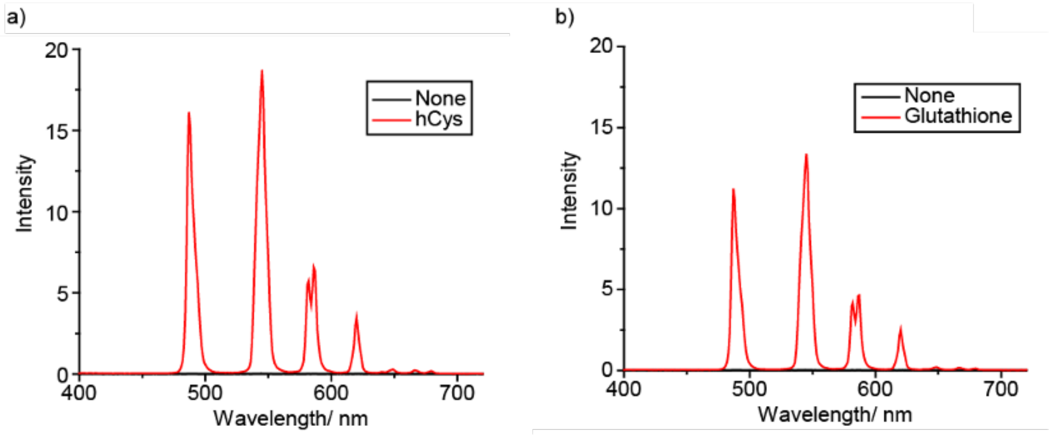

Figure S2. Emission spectra after 16 hours incubation of **Tb.C12** (250 μM) with (a) homocysteine (4 mM) or (b) glutathione (4 mM), compared to the spectra without thiol addition which sits on the baseline. Incubations in water, pH 7.0 at 37 °C, emission spectra (λ_{exc} = 280 nm) recorded after 10-fold dilution into 10 mM HEPES, pH 7.0.

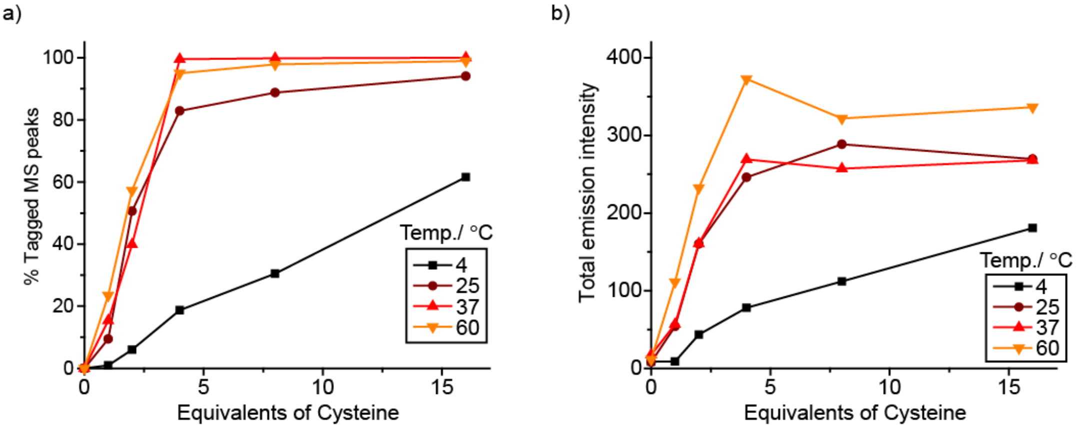

Figure S3. Incubation of **Tb.C12** (250 μM) with different equivalents of cysteine at different temperatures for 16 hours. Reaction completion monitored by a) mass spectra and b) total emission intensity. Incubations conducted in water at pH 7.0, diluted 10-fold into 10 mM HEPES, pH 7.0, for emission spectra (λ_{exc} = 280 nm). Part a is reproduced from Figure 3a of the main text. Four equivalents of cysteine achieved near-complete reaction. Similar reactivity was observed for the Eu(III) complex of **C12**, albeit with a smaller enhancement in quantum yield upon cysteine tagging (Table 1, Figures S4b and S5).

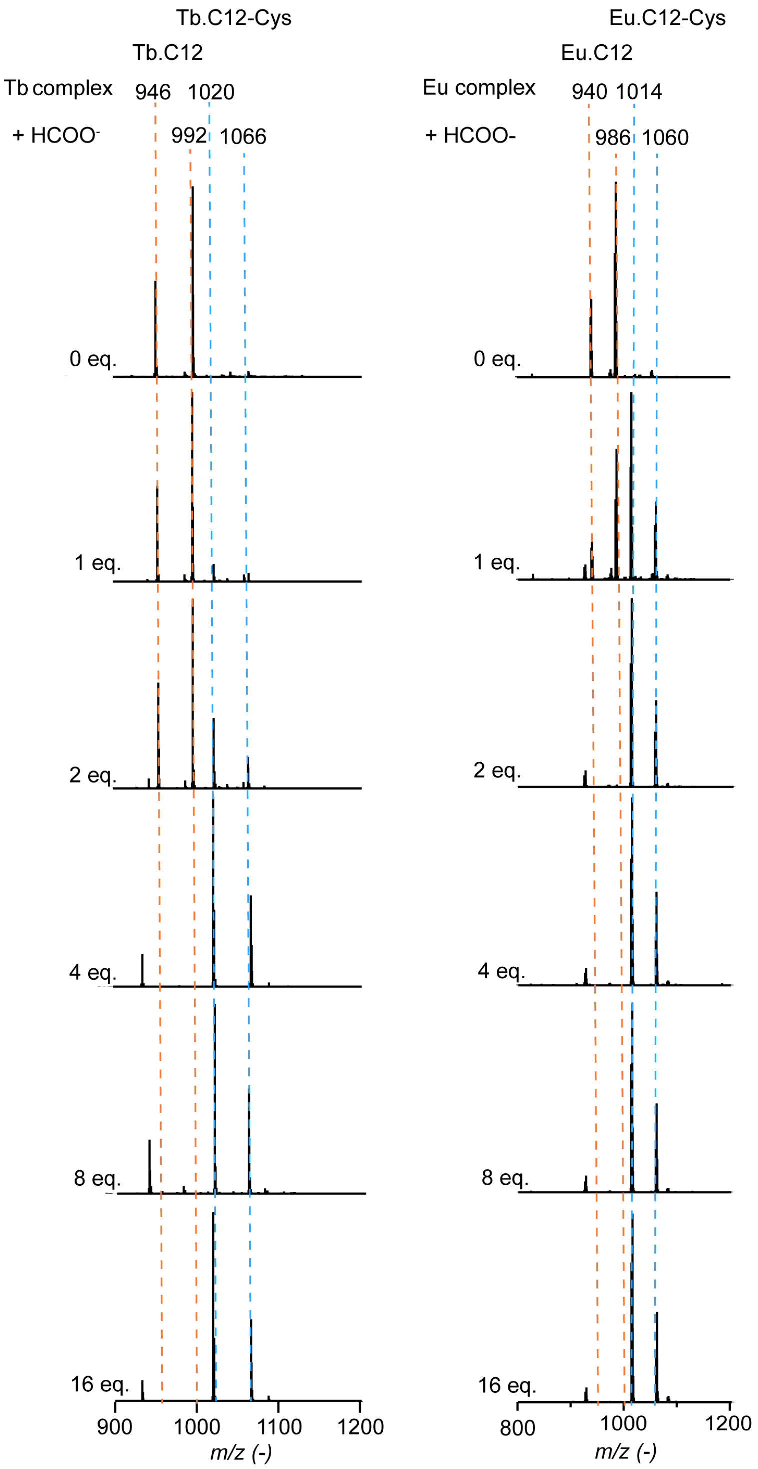

Figure S4. Mass spectra (ESI, negative mode) after incubation of **Tb.C12** or **Eu.C12** (250 μM) with different equivalents of cysteine at 37 °C for 16 hours. Tagging yields were calculated by comparing the intensity of the cysteine-tagged MS peak relative to the untagged peak. (a) Tagging of **Tb.C12**. The m/z values of **Tb.C12**-Cys and untagged **Tb.C12** are 1020 and 946, respectively (the corresponding formate adducts are at m/z = 1066 and 992, respectively) (b) Tagging of **Eu.C12**. The m/z values of **Eu.C12**-Cys and untagged **Eu.C12** are 1014 and 940, respectively (the corresponding formate adducts are at m/z = 1060 and 986).

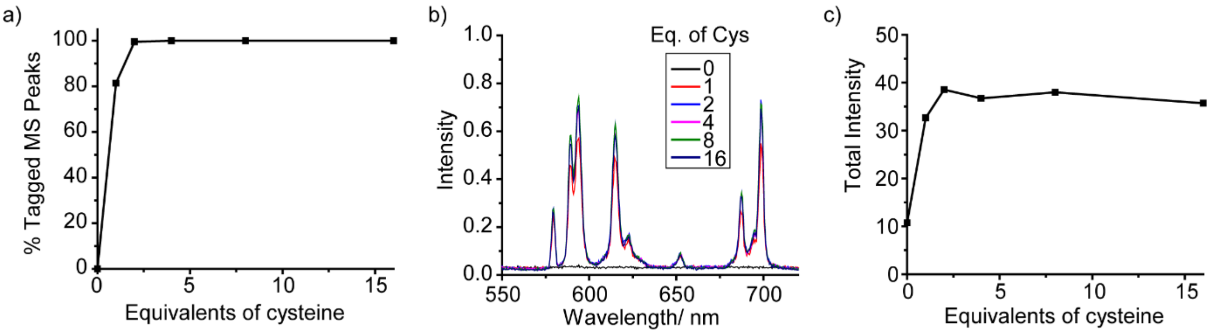

Figure S5. Incubation of **Eu.C12** (250 μM) with different equivalents of cysteine at 37 °C for 16 hours. Reaction completion monitored by a) mass spectra, b) emission spectra and c) total emission intensity. Incubations run in water, pH 7.0, diluted 10-fold into 10 mM HEPES, pH 7.0 for emission spectra (λ_{exc} = 280 nm).

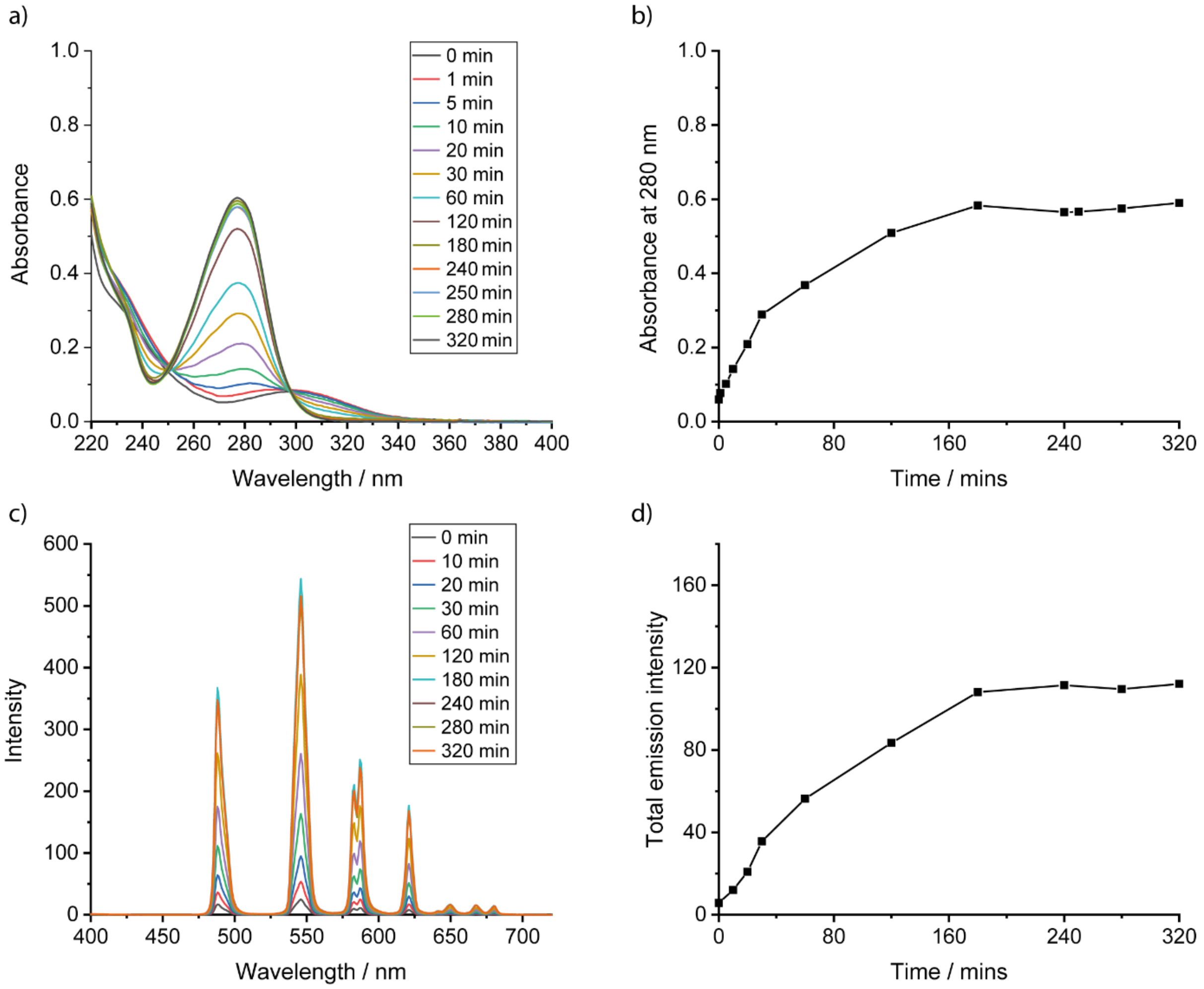

Figure S6. Monitoring the reaction between **Tb.C12** and cysteine. (a) Absorption spectra and (b) absorbance at 280 nm of **Tb.C12** (50 µM) recorded as a function of time following incubation with cysteine (8 equiv.) in 10 mM HEPES, pH 7.0, at 37 °C. (c) Emission spectra and (d) total emission intensity of **Tb.C12** (10 µM) recorded as a function of incubation time with cysteine (8 equiv.) at 37 °C in 10 mM HEPES, pH 7.0 (λ_{exc} = 280 nm).

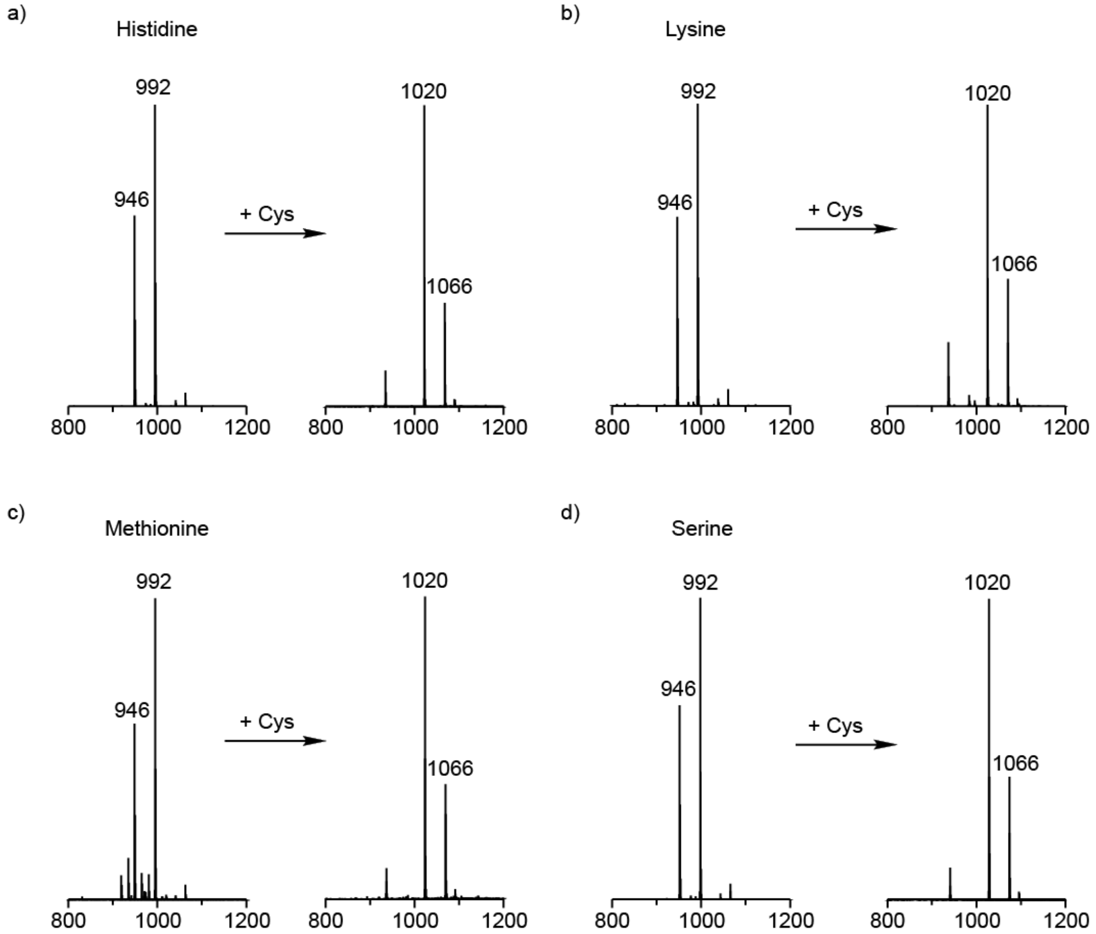

Figure S7. Mass spectra after 24 hour incubation of **Tb.C12** (250 μM) with a) histidine, b) lysine, c) methionine and d) serine (all 4 mM), followed by addition of cysteine (4 mM) and 24 hour incubation in water, pH 7.0 at 37 °C.

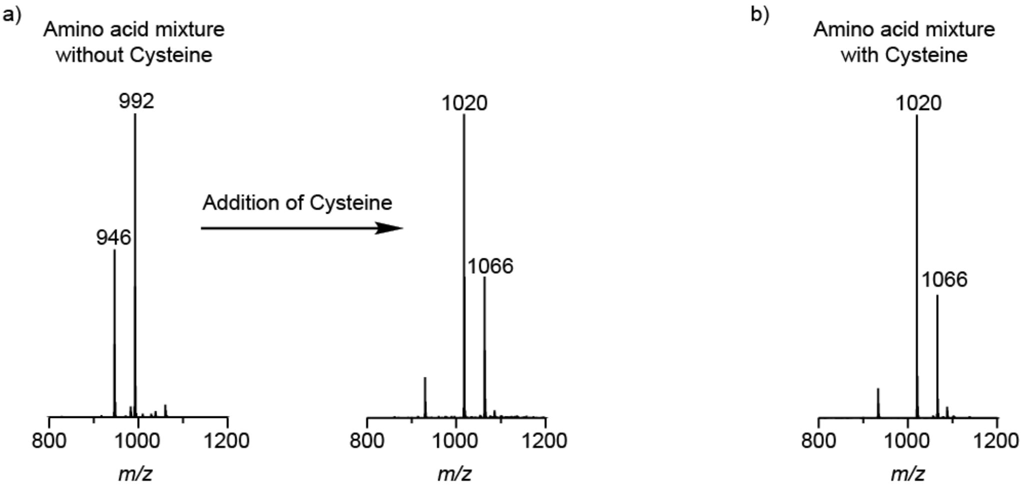

Figure S8. Mass spectra after 24 hour incubation of **Tb.C12** (250 μM) with an amino acid mixture (histidine, arginine, serine, tyrosine, aspartic acid, methionine, asparagine, tryptophan, all 4 mM), with cysteine (4 mM) and without cysteine followed by addition of cysteine (4 mM) and 24 hour incubation in water, pH 7.0, at 37 °C.

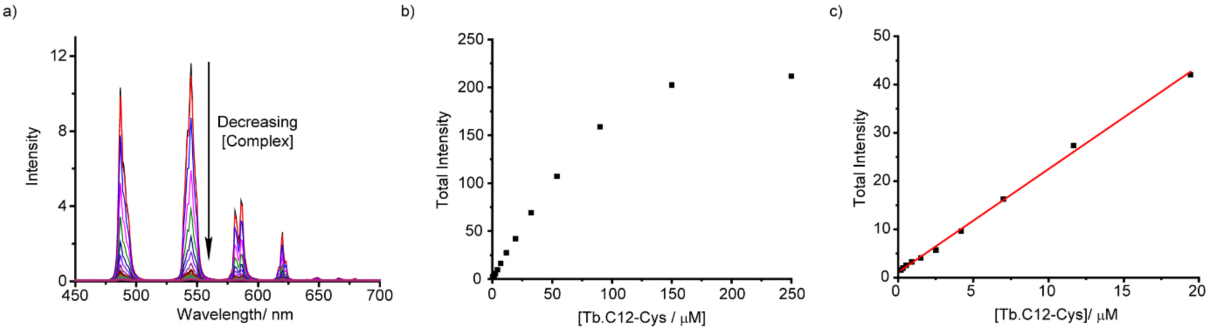

Figure S9. Dilution study of **Tb.C12**-Cys. (a) Emission spectra (λ_{exc} = 280 nm, λ_{em} 400 – 720 nm) of **Tb.C12**-Cys with decreasing concentration in 10 mM HEPES, pH 7.0. Data measured in a 384-well plate reader. (b) Total emission intensity of the spectra in part a. (c) Total emission intensity of **Tb.C12**-Cys measured in the range 0 – 20 μM.

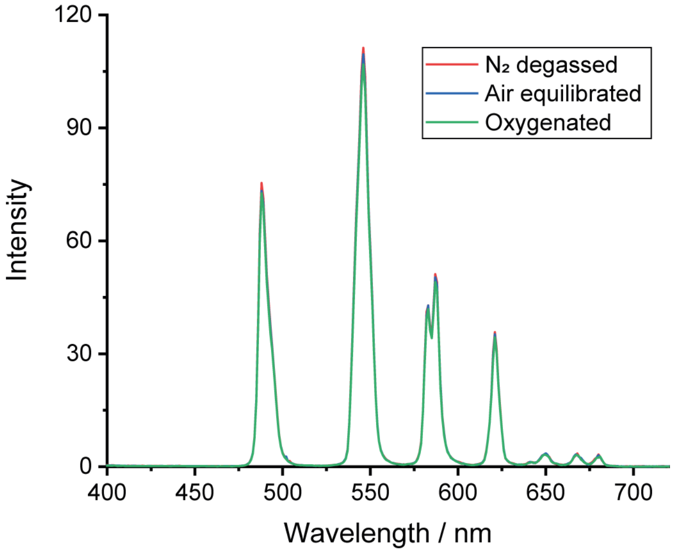

Figure S10. Emission intensity (λ_{exc} = 280 nm, λ_{em} = 450 – 720 nm) of **Tb.C12-Cys** (5 μM) in nitrogen-degassed (5 minutes bubbling), air-equilibrated and oxygenated (5 minutes bubbling) aqueous solution at pH 7. The spectra are indistinguishable within the uncertainty of reproducibility.

a)

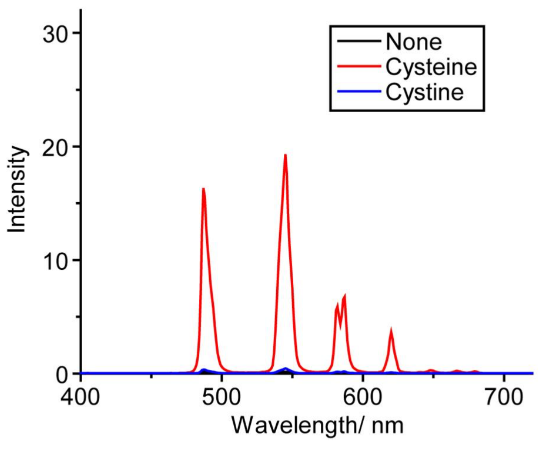

b)

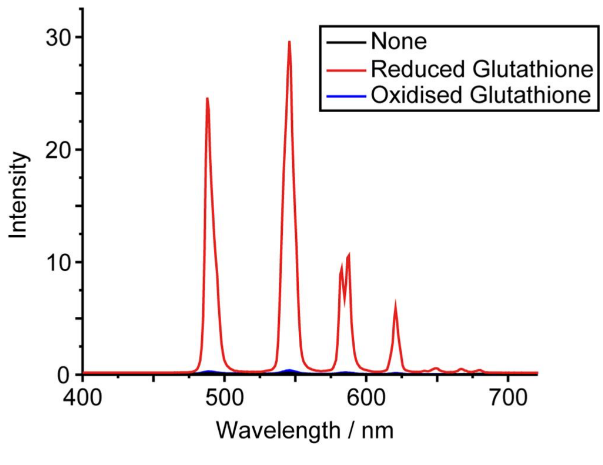

Figure S11. Emission spectra after 16 hours incubation of **Tb.C12** (250 μM) with cysteine, cystine, GSH or GSSG (4 mM). Incubations run in water, pH 7.0, at 37 °C. Emission spectra (λ_{exc} = 280 nm) recorded after 10-fold dilution into 10 mM HEPES, pH 7.0. (a) Cysteine/cystine. (b) Glutathione (oxidised/reduced).

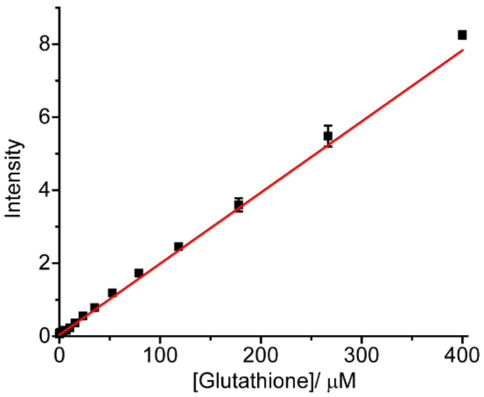

Figure S12. Time-resolved luminescence intensity as a function of glutathione concentration. Data measured 5 minutes after addition of **Tb.C12** (25 μM final concentration) to different concentrations of glutathione in 50 mM Tris-HCl, pH 7.4, using λ_{exc} = 292 – 366 nm, λ_{em} = 510 – 500 nm and an integration time from 60 to 400 μs.

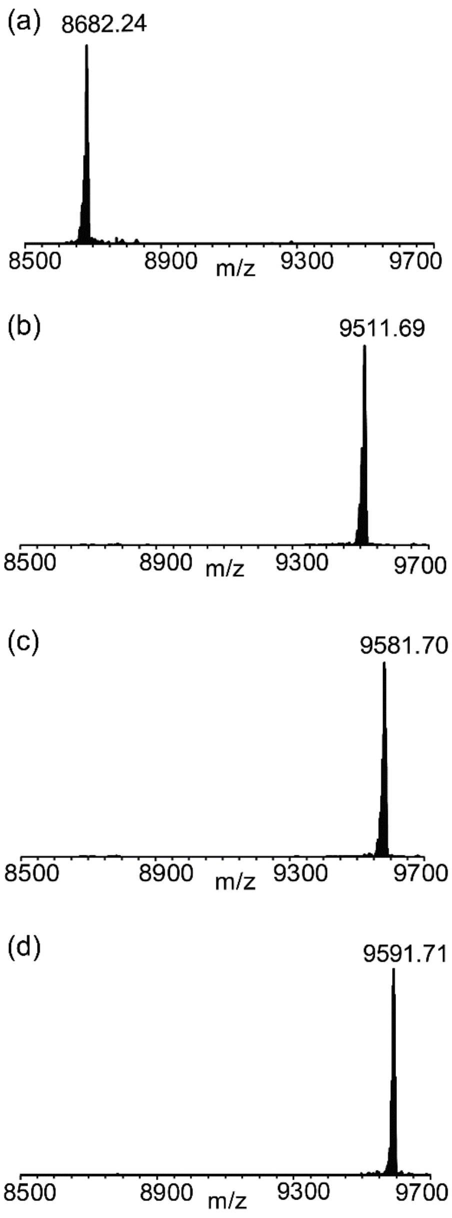

Figure S13. Mass spectra showing complete tagging of uniformly ^{15}N-labelled ubiquitin S57C with the **C12** tag loaded with different lanthanides. (a) Protein without tag. (b) After tagging with **Y.C12**. The expected mass increase is 832.34 Da to 9514.58 Da. (c) After tagging with **Tb.C12**. The expected mass increase is 902.36 Da to 9584.60 Da. (d) After tagging with **Tm.C12**. The expected mass increase is 912.39 Da to 9594.63 Da.

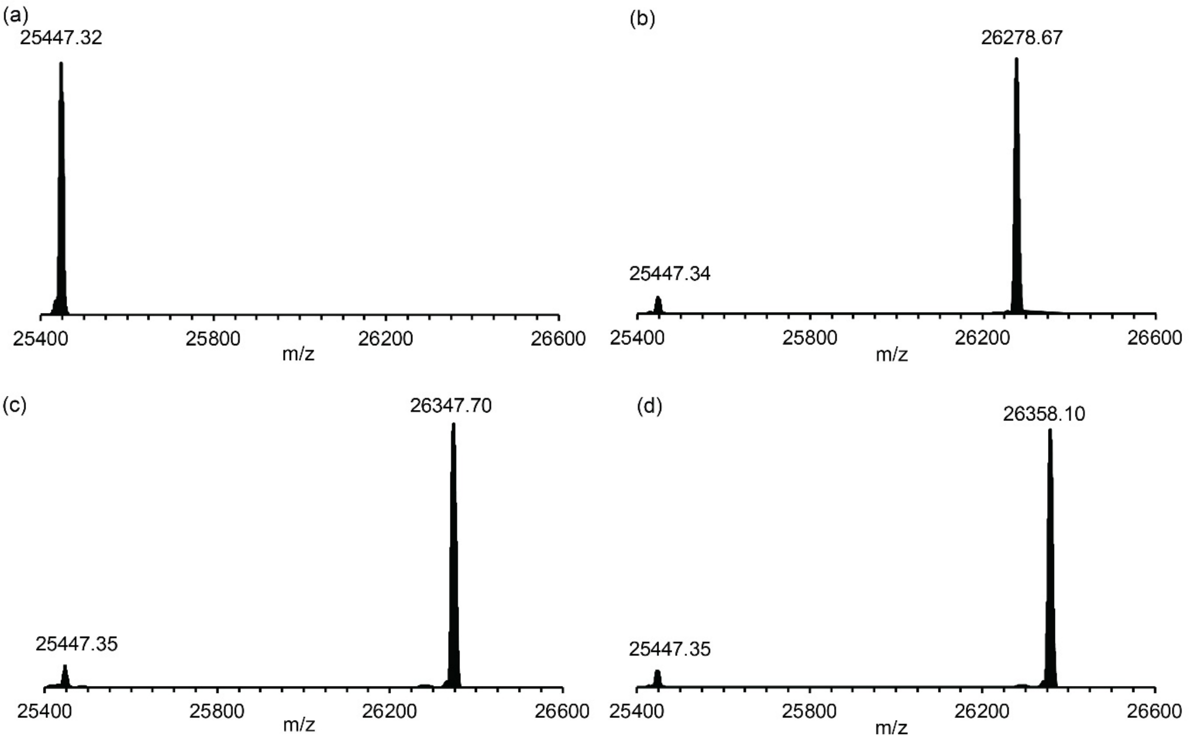

Figure S14. Mass spectra showing complete tagging of uniformly ^{15}N-labelled IMP1 N172C with the **C12** tag loaded with different lanthanoids. (a) Protein without tag. (b) After tagging with **C12**-Y^{3+}. The expected mass increase is 831.86 Da to 26279.18 Da. (c) After tagging with **C12**-Tb^{3+}. The expected mass increase is 901.88 Da to 26349.2 Da. (d) After tagging with **C12**-Tm^{3+}. The expected mass increase is 912.40 Da to 26359.72 Da.

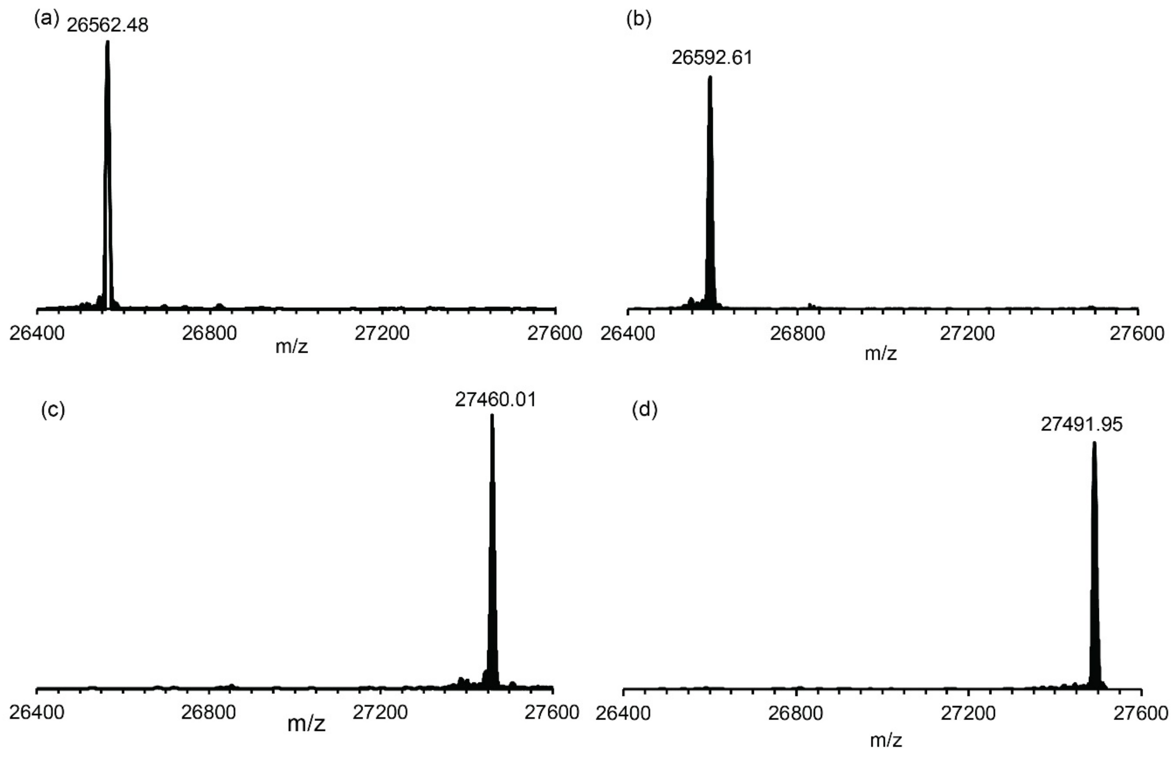

Figure S15. Mass spectra showing complete tagging of ERp29 S114C and G147C mutants with the **Gd.C12** tag. All ERp29 samples also contained the mutant C157S to remove the single naturally occurring cysteine residue in the protein. (a) ERp29 S114C without tag. The calculated mass is 26,561.94 Da. (b) ERp29 G147C without tag. The calculated mass is 26,591.96 Da. (c) Same as part a, but after tagging with **Gd.C12**. Calculated mass 27,462.14 Da. (c) Same as part b, but after tagging with **Gd.C12**. The calculated mass is 27,492.16 Da.

Table S1. Pseudocontact shifts measured in ppm for backbone amide protons of ubiquitin S57C ligated with **Tb.C12** or **Tm.C12**.

Residue	PCS with Tb^{3+}	PCS with Tm^{3+}
Gln 2	-0.248	0.137
Ile 3	-0.225	0.142
Val 5	0.017	-0.014
Lys 6	0.178	-0.107
Thr 7	0.092	-0.056
Leu 8	0.124	-0.074
Thr 9	0.085	-0.046
Lys 11	0.053	-0.034
Thr 12	0.017	
Ile 13	0.013	
Thr 14	-0.099	0.072
Leu 15	-0.185	0.114
Val 17	-0.587	0.519
Lys 29	-0.554	0.421
Ile 30	-0.323	0.252
Asp 32	-0.276	0.216
Lys 33	-0.218	0.169
Glu 34	-0.141	0.111
Gly 35	-0.131	0.110
Ile 36	-0.092	0.089
Asp 39	-0.089	0.095
Gln 40	-0.015	0.040
Gln 41	0.027	0.012
Leu 43	0.467	
Ile 44	0.510	-0.277
Phe 45	1.129	-0.663
Ala 46	0.820	-0.536
Gly 47	0.766	-0.488
Leu 50	1.030	-0.547
Arg 54	-0.390	
Lys 63	-0.100	
Glu 64	-0.029	-0.054
Ser 65	0.167	-0.190
Thr 66	0.386	-0.294
Leu 67	0.258	-0.178
His 68	0.448	-0.260
Val 70	0.237	-0.131
Leu 71	0.148	-0.073
Arg 72	0.132	-0.055
Leu 73	0.140	-0.078
Arg 74	0.078	-0.040
Gly 75	0.089	-0.056
Gly 76	0.117	-0.058

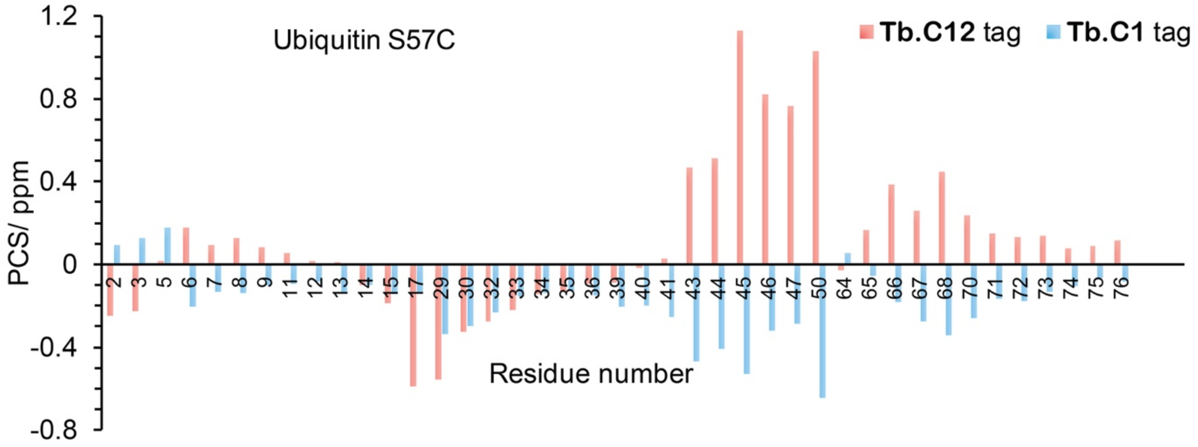

Figure S16. PCSs measured for backbone amide protons of ubiquitin S57C with **Tb.C12** and **Tb.C1** tags[10] versus the amino acid sequence.

Table S2. Pseudocontact shifts measured in ppm for backbone amide protons of IMP-1 N172C ligated with **Tb.C12** or **Tm.C12** tags.

Residue	PCS with Tb^{3+}	PCS with Tm^{3+}
Lys 8	0.110	-0.097
Leu 12	0.013	-0.017
Glu 14		-0.009
Gly 15	0.011	-0.013
Val 18	0.022	-0.06
Thr 20	0.106	-0.097
His 34		-0.093
Gly 35	0.095	-0.113
Leu 36	0.031	-0.078
Val 37	0.028	-0.058
Asp 48	0.074	-0.124
Val 64	0.061	-0.041
Gly 67	0.026	-0.04
Ile 70	0.047	-0.05
Gly 72	0.001	-0.04
Ser 76	0.045	-0.14
Asn 90	0.172	-0.129
Arg 92	0.117	-0.084
Ser 93	0.051	-0.057
Ile 94	0.065	-0.051
Ser 99	-0.249	0.159
Gly 110	-0.002	-0.016
Phe 118	-0.235	0.206
Gly 120	-0.011	0.043
Leu 125	-0.076	0.05
Asn 128	-0.058	0.018
Lys 129	-0.053	0.02
Ile 130	-0.040	0.037
Glu 131	-0.075	0.041
Val 145	-0.288	0.156
Trp 147	-0.117	0.063
Leu 148	-0.049	0.01
Ile 153	-0.057	0.013
Lys 189	-0.073	

Table S3. PCSs measured in ppm for backbone amide protons of IMP-1 N172C ligated with **Tb.C2** or **Tm.C2** tags.

Residue	PCS with Tb^{3+}	PCS with Tm^{3+}
Leu 4	-0.135	0.102
Lys 8	-0.148	0.120
Glu 10	-0.106	0.083
Leu 12	-0.069	0.061
Glu 14	-0.042	0.033
Gly 15	-0.030	0.025
Tyr 17	-0.066	0.058
Val 18	-0.103	0.081
His 19	-0.155	0.125
Thr 20	-0.162	0.127
Phe 22	-0.231	0.178
Glu 24	-0.294	0.219
Val 25	-0.417	0.310
Val 31	-0.389	0.291
Gly 35	-0.222	0.178
Val 37	-0.158	0.135
Val 40	-0.094	0.078
Tyr 45	-0.025	0.023
Leu 46	-0.031	0.034
Ile 47	-0.098	0.088
Asp 48	-0.143	0.125
Thr 49	-0.210	0.177
Thr 52	-0.170	0.136
Asp 55	-0.099	0.079
Thr 61	-0.041	0.033
Val 64	-0.026	0.022
Arg 66	-0.026	0.017
Ile 74	-0.051	0.054
Ser 76	-0.283	0.247
His 77	-0.465	0.409
Ser 82	-0.537	0.435
Thr 83	-0.373	0.302
Gly 84	-0.256	0.212
Gly 85	-0.138	0.119
Asn 90	0.042	-0.030
Arg 92	0.037	-0.028
Ser 93	0.051	-0.039
Ile 94	0.038	-0.025
Ala 114	0.174	-0.123
Ser 117	0.234	-0.182
Phe 118	0.297	-0.225
Ser 119	0.354	-0.266
Tyr 123	0.190	-0.131
Trp 124	0.145	-0.106

Leu 125	0.076	-0.052
Asn 128	0.067	-0.048
Lys 129	0.044	-0.029
Ile 130	0.040	-0.026
Glu 131	0.030	-0.016
Val 132	0.102	-0.069
Phe 133	0.070	-0.028
Tyr 134	0.320	-0.217
Val 144	-0.351	0.336
Val 145	-0.025	0.059
Leu 148	-0.018	0.025
Ile 153	-0.032	0.030
Leu 154	-0.080	0.073
Ala 180	0.167	-0.092
Gly 188	0.053	-0.035
Lys 189	0.031	-0.016
Lys 191	-0.036	0.027
Leu 192	-0.062	0.053
Val 194	-0.174	0.145
Gly 201	-0.176	0.141
Ser 204	-0.089	0.073
Leu 210	-0.158	0.134
Glu 211	-0.148	0.118
Ala 213	-0.355	0.281
Gly 216	-0.454	0.354

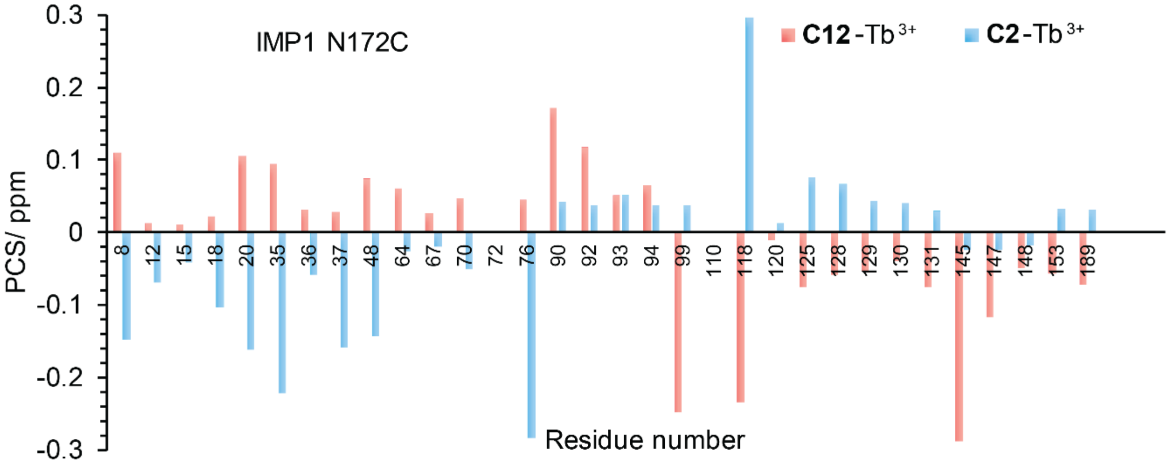

Figure S17. Plots of the PCSs measured for backbone amide protons of IMP1 N172C with **Tb.C12** tag and **Tb.C2** tag versus the amino acid sequence.

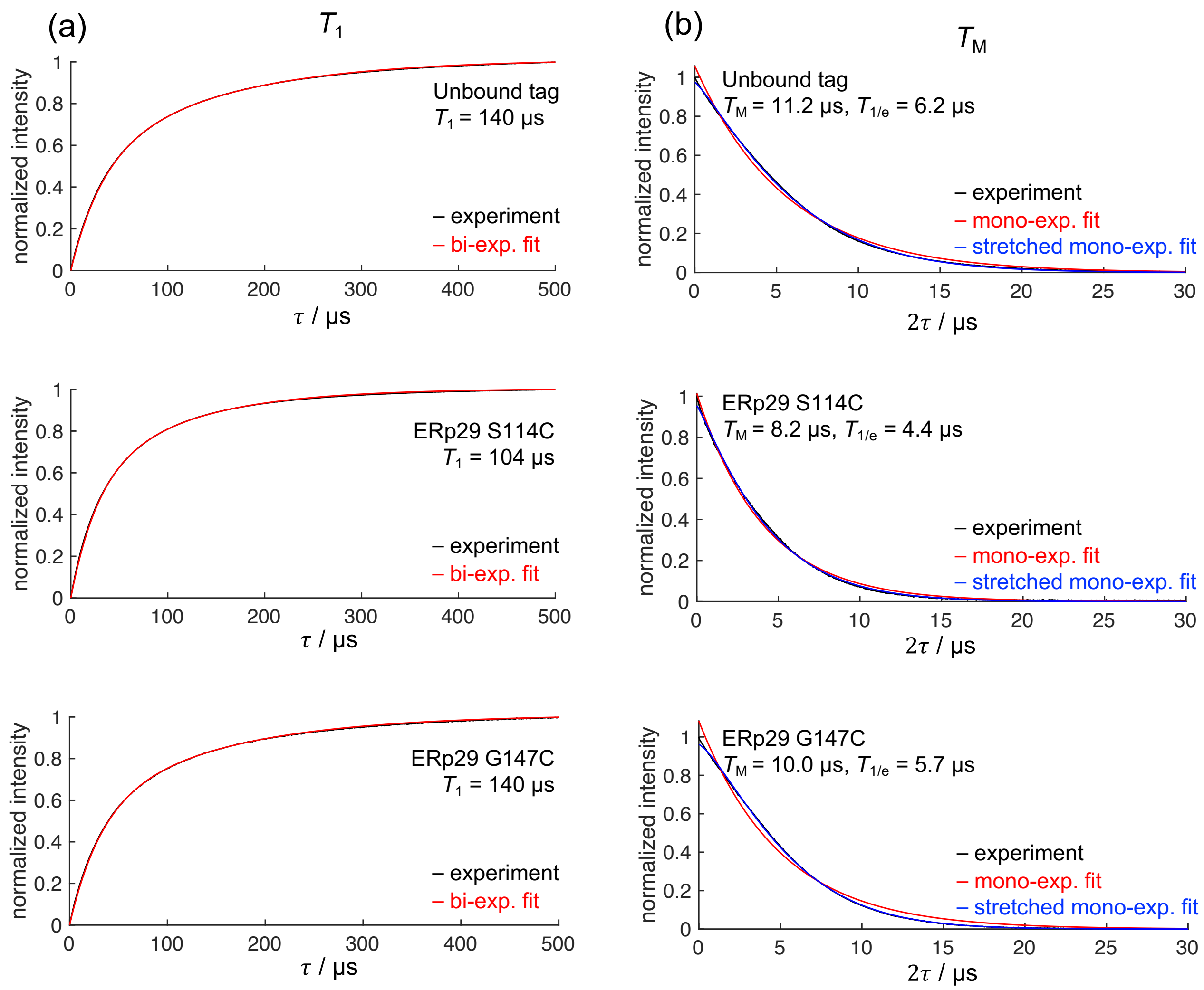

Figure S18. EPR characterisation of the **Gd.C12** tag, free and bound to ERp29 S114C and ERp29 G147C and associated fits. All fits were performed using a Levenberg–Marquardt least squares fitting in MATLAB. The modelled time constants are annotated in each figure. (a) T_1 inversion recovery measurements (black) were fit with a biexponential decay function of the form [Aexp($-t/\tau_1$) + Bexp($-t/\tau_2$)] (red). (b) Spin-echo decay measurements of the phase memory time T_M (black) with corresponding mono-exponential fit of the data in red stretched function fit $\exp[-(2t/\tau)^a]$ in blue. The $T_{1/e}$ value is the time taken for the signal to reach 1/e of its initial value, where T_M is close to $2T_{1/e}$.[13]

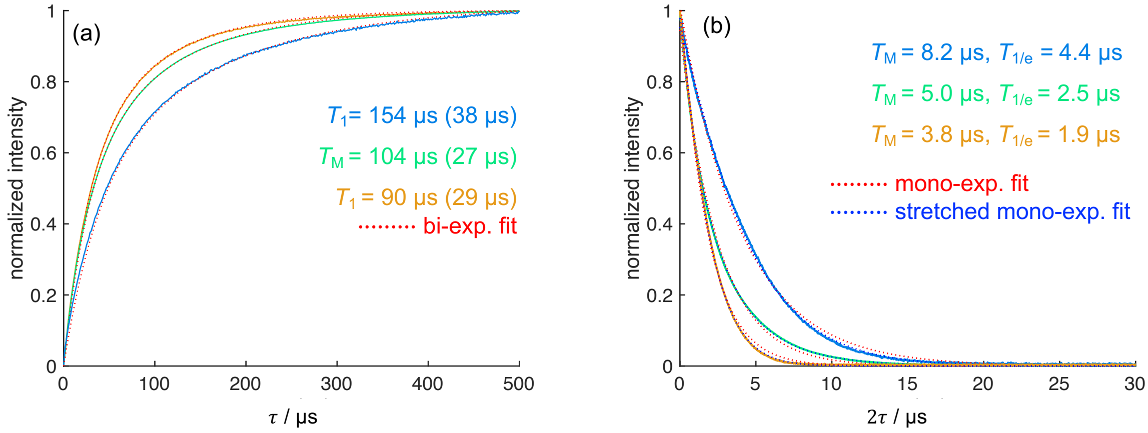

Figure S19. Inversion recovery (a) and spin-echo decay (b) time constants as a function of hydrogen–deuterium exchange. Samples of ERp29 S114C tagged with **Gd.C12** were buffer-exchanged into D_2O at 4 °C as follows: 50 µL of the protein in H_2O-based buffer was diluted 10-fold with D_2O-based buffer and reconcentrated using an Amicon ultrafiltration device with a molecular weight cut-off of 10 kDa. This was repeated five times. The exchange protocol was completed within three hours and the sample frozen for EPR measurements (orange curve). Repeating the buffer exchange an additional five times yielded the EPR data shown in green. Leaving this sample in 1 mL deuterated buffer for an additional 24 hours at room temperature followed by 10 more buffer exchange steps yielded the blue curve. Based on these results, a simplified exchange protocol was used for ERp29 G147C tagged with **Gd.C12**, which was buffer-exchanged into D_2O by 5 repeats of 10-fold dilution, followed by 24 hours incubation at room temperature. This sample yielded a spin-echo decay time constant of 8.2 µs. Bi/mono-exponential and stretched exponential decay fits for all three curves are shown using red and blue dotted traces respectively.

Table S4. Parameters used to fit the relaxation data of the **Gd.C12** tag.[a]

	T_1 - τ_1 (µs)	T_1 - τ_2 (µs)	T_M (µs) monoexp.	T_M (µs) / stretch factor a	$T_{1/e}$ (µs)
Unbound tag	139.7	32.1	11.2	12.5 / 1.21	6.2
ERp29 S114C	104.2	27.4	8.2	8.9 / 1.14	4.4
ERp29 G147C	139.9	31.4	10.0	11.7 / 1.33	5.7

[a] The T_1 relaxation data were fitted using the biexponential function [$A\exp(-t/\tau_1) + B\exp(-t/\tau_2)$]. The T_M relaxation time was fitted by the mono-exponential decay function $\exp[-(2t/\tau)]$ and are compared against a stretched exponential decay fit $\exp[-(2t/\tau)^a]$ in the subsequent column along with the $T_{1/e}$ time in the last column.

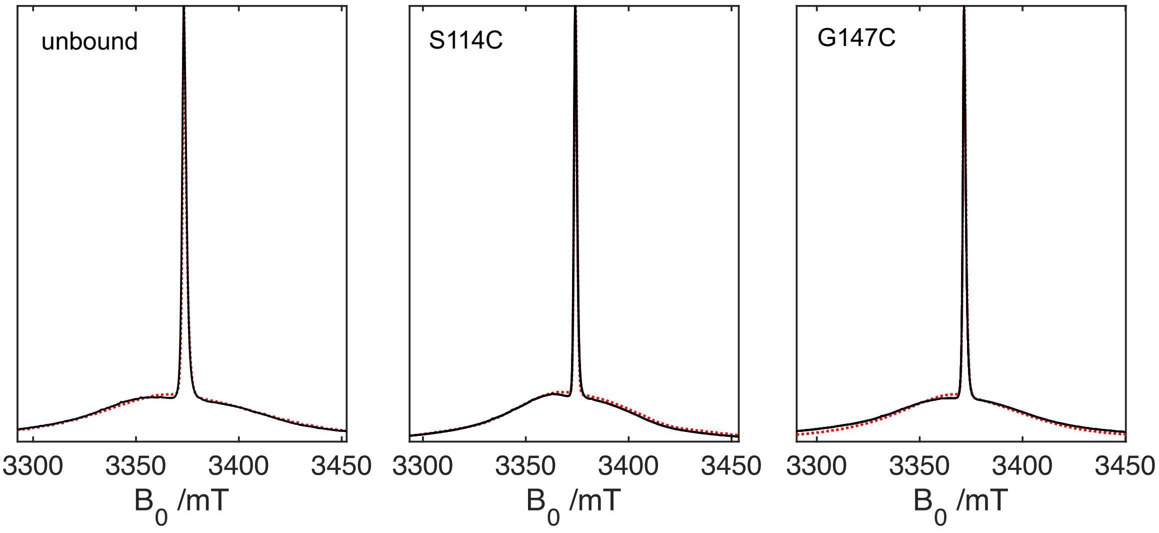

Figure S20. Lineshape fits of **Gd.C12**, unbound and bound to ERp29 S114C and ERp29 G147C, respectively. The experimental data are shown in black and simulated spectra are represented by the dotted red trace. The simulations were performed using the EasySpin *pepper* algorithm.[14] The fitting procedure employed the model by Clayton *et al.*,[15] which was based on the original description by Raitsimring *et al.*[16] of more accurate calculations for *D*-strains on the order of the ZFS tensor. The *D*-value distribution was modelled as a bimodal distributions centred about $\pm|D|$ with width σ_D and weighting of the positive and negative peaks by a ratio of P^{D+}/P^{D-}. The *E*/*D* ratio was modelled by the polynomial $P(E/D) = E/D - 2(E/D)^2$. The fitting was performed as a Monte Carlo simulation, using randomly generated parameter sets (D, σ_D, P^{D+}/P^{D-} and E/D) as starting points for the simulations, with the final spectrum calculated as a weighted sum according to the modelled *D* and *E*/*D* distributions. A finite linewidth (*lw*) was included to model the central sharp feature. The fits shown are the lowest RMSD simulations with a sample size of 200, constraining the search parameters based on previously reported values for similar tags.[15]

Table S5. Parameters used to fit the line shape of the **Gd.C12** tag.

	g-value	$\|D\|$ /MHz	σ_D/MHz	*P(D+)/P(D-)*	*lw* (mT)
Unbound tag	1.9917	810.6	224.3	3.2	0.56
ERp29 S114C	1.9920	555.4	100.5	2.7	1.2
ERp29 G147C	1.9917	653.0	195.8	3.7	0.86

	g value	$\|D\|$/MHz	σ_D/MHz	$P(D+)/P(D-)$	*lw*/mT
Unbound tag	1.9917	810.6	224.3	3.2	0.56
ERp29 S114C	1.9920	555.4	100.5	2.7	1.2

ERp29 G147C	1.9917	653.0	195.8	3.7	0.86

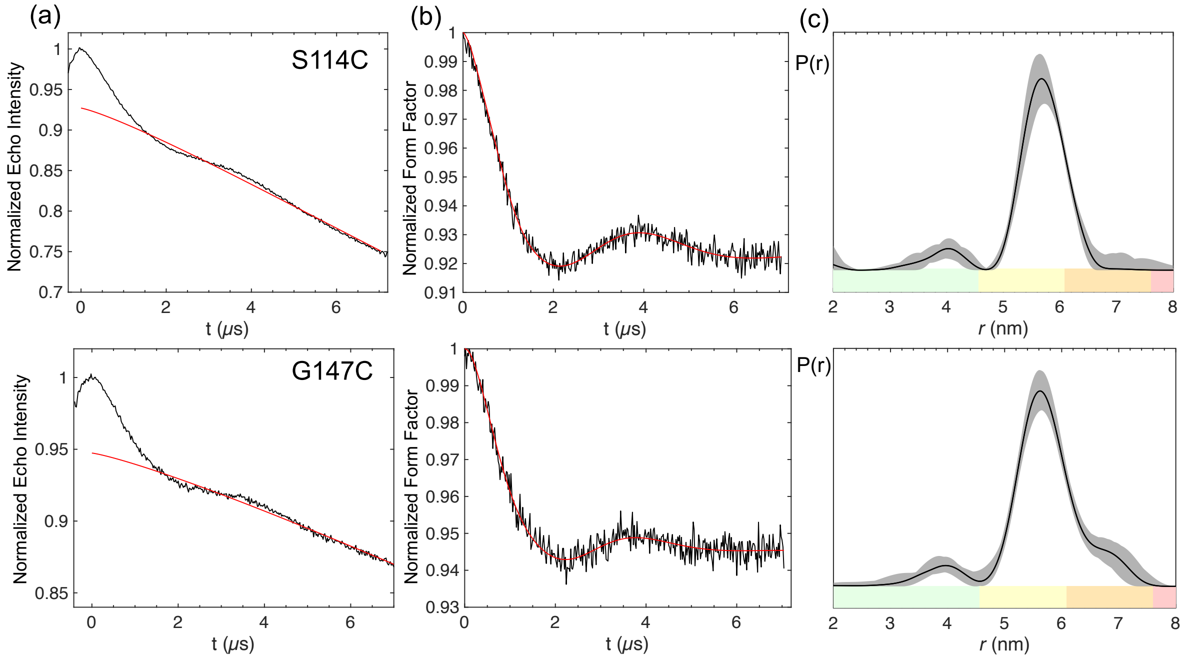

Figure S21. DEER experiments of ERp29 S114C (top panel) and ERp29 G147C (bottom panel) tagged with the **Gd.C12** tag. (a) Raw DEER data acquired in 19 h experiments. The red line shows the background fit, which is subtracted from the raw data to give the form factor in (b). The form factors in (b) are also shown in Figure 8a of the main text. (c) Distance distribution derived from the data in (b) using DeerAnalysis2019. Reliability ranges are identified by different colours: green - the shape of the distance distribution is reliable, yellow - the maximum of the distribution and its width are reliable, orange - the mean distance is reliable, red - long-range distances may be observable but cannot be quantified. The grey regions represent the uncertainty range of the data, indicating the range of alternative distributions obtained by varying the parameters of the background correction using the validation tool in the DeerAnalysis2018 software package. Parameter ranges used for the validations: white noise 0–1.5, background start $0.2*t_{max}$–$0.6*t_{max}$, background dimension 3.0, regularisation parameter 630.

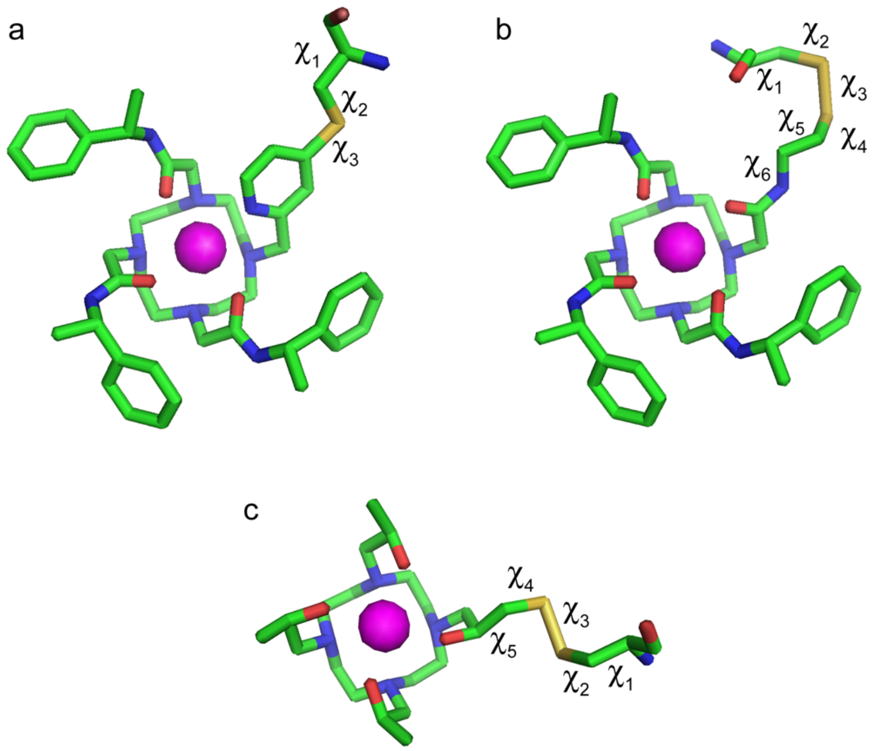

Figure S22. Tag structures and generation of rotamer libraries. The rotamer libraries were established by modeling the **C12**, **C1** and **C7** tags onto the crystal structure of human ERp29 (PDB 2QC7)[17] with the C-terminal domain removed to account for the flexibility of the linker between the N- and C-terminal domains.[18] The dihedral angles identified in the structures of parts a and b were varied in random combinations using the program PyParaTools. Conformations generating steric clashes with the protein were excluded. (a) Heavy-atom representation of a cysteine residue with the **Gd.C12** tag. The lanthanoid ion is indicated by a magenta sphere. The dihedral angles varied to create rotamer libraries are indicated. To generate the rotamer libraries, the dihedral angle χ_1 was allowed to vary by ±30° around the staggered rotamers and χ_2 varied by ±30° around the 90° and -90° values, while the angle χ_3 was varied completely randomly. (b) Same as part a, but for the **Gd.C1** tag. The dihedral angle χ_1 was allowed to vary by ±30° around the staggered rotamers, χ_2 varied by ±30° around the 90° and -90° values, and χ_3, χ_4, χ_5 and χ_6 were varied randomly. (c) Same as part a, but for the **Gd.C7** tag. The dihedral angles χ_1–χ_5 were varied as for the **Gd.C1** tag.

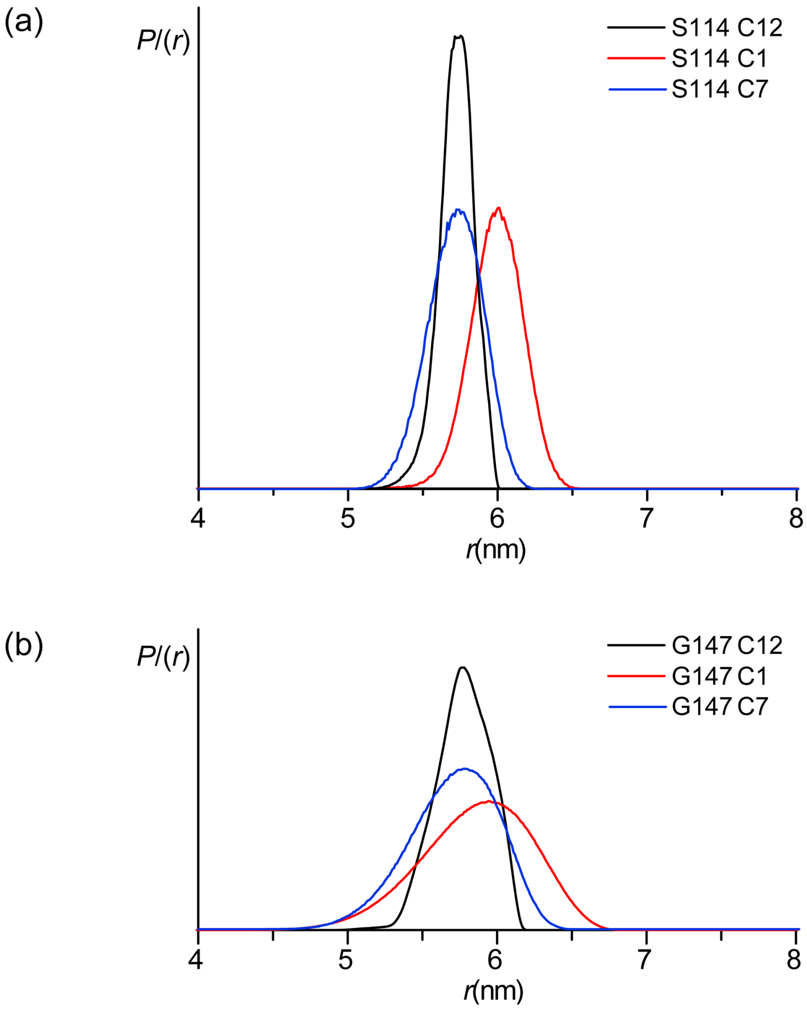

Figure S23. Gd^{3+}–Gd^{3+} distance distributions calculated with the program PyParaTools using the rotamer libraries and angle variations described in Figure S22. (a) ERp29 S114C tagged with **Gd.C12** (black), **Gd.C1** (red) and **Gd.C7** (blue). (b) Same as part a, but for ERp29 G147C. Distance distributions calculated for **Gd.C8** (the enantiomer of **Gd.C7**) were practically indistinguishable from those of **Gd.C7**.

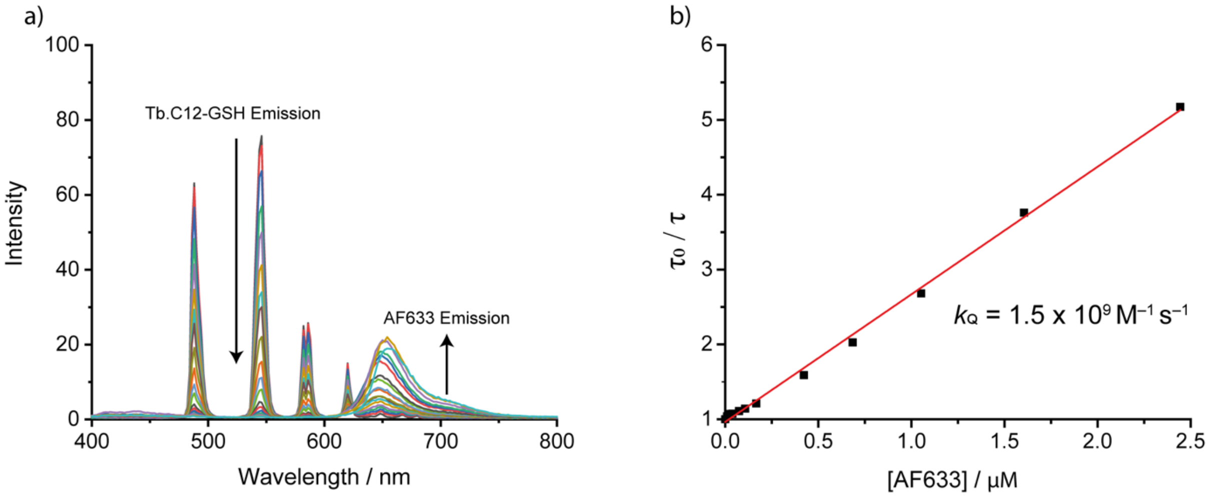

Figure S24. FRET between **Tb.C12-GSH** (donor) and freely diffusing AF633 (acceptor). (a) Decrease in emission intensity of **Tb.C12-GSH** (1 μM) and concomitant increase in AF633 emission intensity (centred at 650 nm) with increasing concentration of AF633 (0 – 2.5 μM), using λ_{exc} = 282 nm and λ_{em} = 400 – 800 nm. Data measured in 50 mM Tris-HCl, pH 7.4, 50 mM NaCl, 5% DMSO. (b) Stern-Volmer plot showing a linear increase in the τ_0/τ ratio (at 546 nm) with increasing AF633 concentration, according to the Stern-Volmer equation

$$\frac{\tau_0}{\tau} = 1 + k_Q\tau_0[\mathrm{Q}] \tag{3}$$

where τ_0 = emission lifetime in the absence of quencher (1109 μs), τ = emission lifetime at given concentration of quencher, k_Q = quenching rate constant, Q = quencher (AF633).

Quenching of the Tb(III) emission in the presence of AF633 results in shortening of the emission lifetime, τ . The quenching rate constant, k_Q = 1.5 x 10^9 M^{-1} s^{-1}.

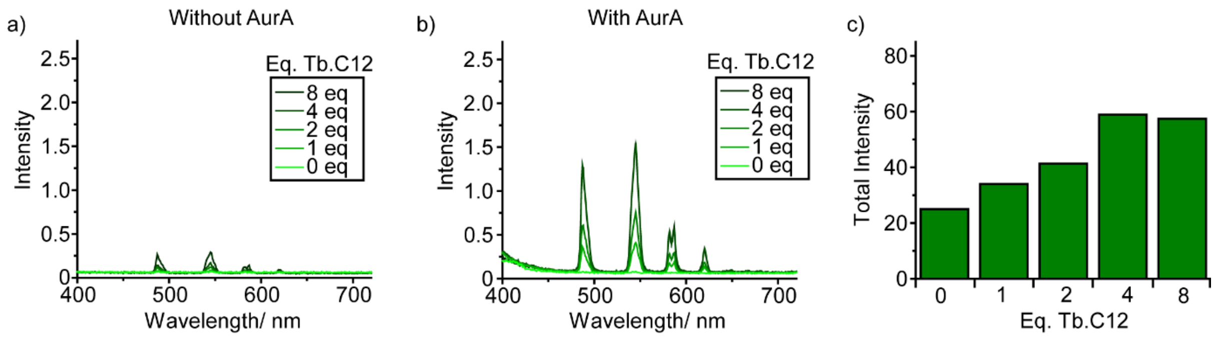

Figure S25. Emission spectra (λ_{exc} = 280 nm) after incubation of Aurora A with different equivalents of **Tb.C12**. Incubation was at 4 °C for 18 hours in 50 mM Tris-HCl, pH 7.4, 50 mM NaCl, 1.8% DMSO. (a) Control without Aurora A. (b) 15 µM Aurora A. (c) Total emission intensity of the data in part b. The data suggest that quantitative tagging was achieved with 4 equivalents of **Tb.C12**.

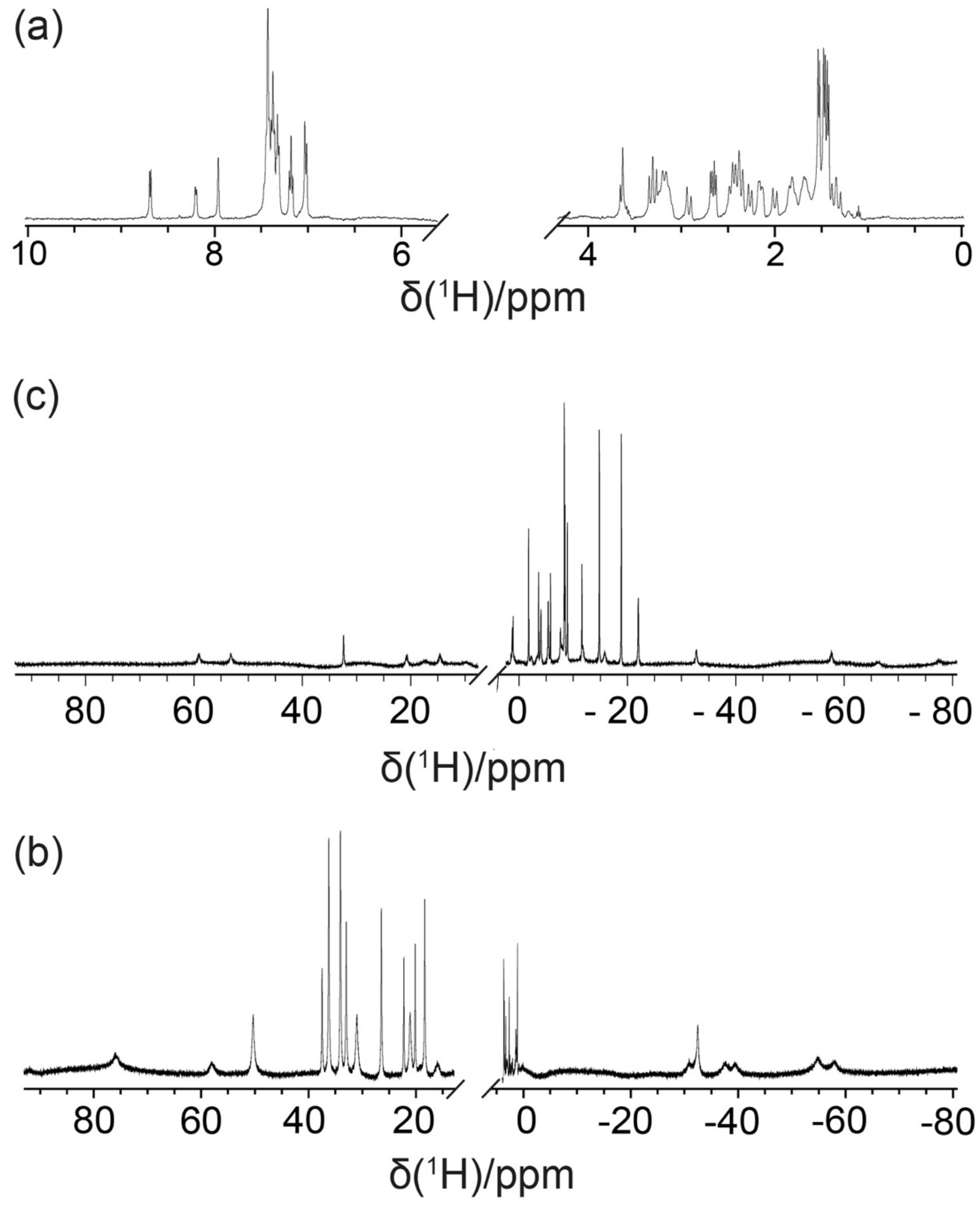

Figure S26. 1D ^1H NMR spectra of the **Ln.C12** tag complexed with either (a) Y^{3+}, (b) Tm^{3+} or (c) Tb^{3+}. The spectra were recorded in 95% D_2O/5% H_2O solution at 25 °C on a 400 MHz NMR spectrometer, using presaturation to suppress the residual HDO resonance. No solute signals were observable in the central regions omitted from the plots.

References

1. K. L. Gempf, S. J. Butler, A. M. Funk and D. Parker, *Chem. Commun.* **2013**, *49*, 9104–9106.
2. L. de la Cruz, T. H. D. Nguyen, K. Ozawa, J. Shin, B. Graham, T. Huber and G. Otting, *J. Am. Chem. Soc.* **2011**, *133*, 19205–19215.
3. T. J. Carruthers, P. D. Carr, C.-T. Loh, C. J. Jackson and G. Otting, *Angew. Chem. Int. Ed.* **2014**, *53*, 14269–14272.
4. A. P. Welegedara, A. Maleckis, R. Bandara, M. C. Mahawaththa, I. D. Herath, Y. J. Tan, A. Giannoulis, D. Goldfarb, G. Otting and T. Huber, *ChemBioChem* **2020**, *22*, 1840–1848.
5. M. A. Apponyi, K. Ozawa, N. E. Dixon and G. Otting, in *Structural Proteomics: High-Throughput Methods*, eds. B. Kobe, M. Guss and T. Huber, Humana Press, Totowa, NJ, **2008**, pp. 257–268.
6. P. S. C. Wu, K. Ozawa, S. P. Lim, S. G. Vasudevan, N. E. Dixon and G. Otting, *Angew. Chem. Int. Ed.* **2007**, *46*, 3356-3358.
7. A.-M. Catanzariti, T. A. Soboleva, D. A. Jans, P. G. Board and R. T. Baker, *Protein Sci.* **2004**, *13*, 1331–1339.
8. C. A. Dodson and R. Bayliss, *J. Biol. Chem.* **2012**, *287*, 1150–1157.
9. C. A. Dodson, *Methods Mol. Biol.* **2017**, *1586*, 251–264.
10. B. J. G. Pearce, S. Jabar, C.-T. Loh, M. Szabo, B. Graham and G. Otting, *J. Biomol. NMR* **2017**, *68*, 19–32.
11. R. Bayliss, T. Sardon, I. Vernos and E. Conti, *Mol. Cell* **2003**, *12*, 851–862.
12. F. C. Rowan, M. Richards, R. A. Bibby, A. Thompson, R. Bayliss and J. Blagg, *ACS Chem. Biol.* **2013**, *8*, 2184–2191.
13. H. Yagi, D. Banerjee, B. Graham, T. Huber, D. Goldfarb and G. Otting, *J. Am. Chem. Soc.* **2011**, *133*, 10418–10421.
14. S. Stoll and A. Schweiger, *J. Magn. Reson*. **2006**, *178*, 42–55.
15. J. A. Clayton, K. Keller, M. Qi, J. Wegner, V. Koch, H. Hintz, A. Godt, S. Han, G. Jeschke, M. S. Sherwin and M. Yulikov, *Phys. Chem. Chem. Phys.* **2018**, *20*, 10470–10492.
16. A. M. Raitsimring, A. V. Astashkin, O. G. Poluektov and P. Caravan, *Appl. Magn. Reson.* **2005**, *28*, 281.

17. N. N. Barak, P. Neumann, M. Sevvana, M. Schutkowski, K. Naumann, M. Malešević, H. Reichardt, G. Fischer, M. T. Stubbs and D. M. Ferrari, *J. Mol. Biol.* **2009**, *385*, 1630–1642.
18. E. H. Abdelkader, M. D. Lee, A. Feintuch, M. R. Cohen, J. D. Swarbrick, G. Otting, B. Graham and D. Goldfarb, *J. Phys. Chem. Lett.* **2015**, *6*, 5016–5021.
